# Characterization of MdpS: an in-depth analysis of a MUC5B-degrading protease from *Streptococcus oralis*

**DOI:** 10.3389/fmicb.2024.1340109

**Published:** 2024-01-18

**Authors:** Fredrik Leo, Rolf Lood, Kristina A. Thomsson, Jonas Nilsson, Gunnel Svensäter, Claes Wickström

**Affiliations:** ^1^Department of Oral Biology and Pathology, Faculty of Odontology, Malmö University, Malmö, Sweden; ^2^Genovis AB, Kävlinge, Sweden; ^3^Department of Clinical Sciences Lund, Division of Infection Medicine, Faculty of Medicine, Lund University, Lund, Sweden; ^4^Proteomics Core Facility, Sahlgrenska Academy at the University of Gothenburg, Gothenburg, Sweden

**Keywords:** MUC5B, *Streptococcus oralis*, O-glycosylation, mucin degradation, serine protease, oral biofilm

## Abstract

Oral biofilms, comprising hundreds of bacteria and other microorganisms on oral mucosal and dental surfaces, play a central role in oral health and disease dynamics. *Streptococcus oralis*, a key constituent of these biofilms, contributes significantly to the formation of which, serving as an early colonizer and microcolony scaffold. The interaction between *S. oralis* and the orally predominant mucin, MUC5B, is pivotal in biofilm development, yet the mechanism underlying MUC5B degradation remains poorly understood. This study introduces MdpS (Mucin Degrading Protease from *Streptococcus oralis*), a protease that extensively hydrolyses MUC5B and offers an insight into its evolutionary conservation, physicochemical properties, and substrate- and amino acid specificity. MdpS exhibits high sequence conservation within the species and also explicitly among early biofilm colonizing streptococci. It is a calcium or magnesium dependent serine protease with strict physicochemical preferences, including narrow pH and temperature tolerance, and high sensitivity to increasing concentrations of sodium chloride and reducing agents. Furthermore, MdpS primarily hydrolyzes proteins with O-glycans, but also shows activity toward immunoglobulins IgA1/2 and IgM, suggesting potential immunomodulatory effects. Significantly, MdpS extensively degrades MUC5B in the N- and C-terminal domains, emphasizing its role in mucin degradation, with implications for carbon and nitrogen sequestration for *S. oralis* or oral biofilm cross-feeding. Moreover, depending on substrate glycosylation, the amino acids serine, threonine or cysteine triggers the enzymatic action. Understanding the interplay between *S. oralis* and MUC5B, facilitated by MdpS, has significant implications for the management of a healthy eubiotic oral microenvironment, offering potential targets for interventions aimed at modulating oral biofilm composition and succession. Additionally, since MdpS does not rely on O-glycan removal prior to extensive peptide backbone hydrolysis, the MdpS data challenges the current model of MUC5B degradation. These findings emphasize the necessity for further research in this field.

## Introduction

Oral biofilms harbor complex communities of commensal bacteria that colonize mucosal and dental surfaces within the oral cavity, and play a significant role in both healthy and diseased states (Marsh, 2010). Biofilms develop naturally, especially on teeth, and benefit the host by preventing colonization by exogenous species (Marsh, 1994). Among the diverse array of bacterial species within these biofilms, *Streptococcus oralis* emerges as a prominent member (Teles et al., 2012). As part of the *Streptococcus mitis* Group, *S. oralis* is a key constituent of the oral microbiota, influencing the intricate equilibrium of oral ecosystems (Lamont et al., 2018). Its multifaceted abilities, including surface adhesion, communication with other microorganisms, and interaction with host components, contribute to its pivotal role in the establishment and maintenance of oral biofilms (Nobbs et al., 2009). *S. oralis* is particularly recognized for its involvement in the initial stages of oral biofilm formation (Nyvad and Kilian, 1990; Diaz et al., 2006). During the early phases of colonization, this bacterium adheres to oral surfaces, such as teeth, and forms microcolonies that serve as scaffolds for subsequent microbial attachment (Diaz et al., 2006; Nobbs et al., 2009). This process is facilitated by the interaction between bacterial surface adhesins and host-derived molecules, such as salivary proteins (Nobbs et al., 2009). Notably, one such host component that influences the colonization of *S. oralis* is mucin, specifically MUC5B.

MUC5B is a gel-forming mucin, prevailing as the predominant glycoprotein found in saliva (Wickström et al., 1998). Oral and respiratory tract MUC5B is produced by mucus cells in the submucosal gland acini and by goblet cells in the airway epithelium (Okuda et al., 2019). With its high molecular weight, MUC5B plays a key role in maintaining the mucosal barrier and promoting clearance of pathogens (Wickström and Svensäter, 2008; Zaretsky and Wreschner, 2013; Frenkel and Ribbeck, 2015). Recently, it was demonstrated that several subclasses of *S. oralis* possess specific surface molecules, such as adhesins, that enable the bacteria to recognize and adhere to the glycosylated structures on MUC5B (Chahal et al., 2022). These interactions could have great implications for the development and maintenance of oral biofilms. In the context of oral biofilm development, the hydrolysis of MUC5B glycans by various streptococci has received considerable attention as regards its impact on the microbial succession (van Der Hoeven et al., 1990). However, the mechanisms of the proteolytic degradation of MUC5B have been mainly disregarded.

Mucin degradation is an essential function for gastrointestinal bacteria to sequester carbon and nitrogen sources for growth (Derrien et al., 2004). The steady pace of accumulating findings in this young research field was broken when the identification of new types of mucin degrading enzymes led to a significantly increased publication rate (Shon et al., 2021). Not only did the enzymes hydrolyze O-glycan monosaccharides and the protein backbone at Lys/Arg-residues, novel O-glycoproteases and glycanases relied on O-glycan dependent and disaccharide hydrolysis, respectively (Crouch et al., 2020; Shon et al., 2021). Because of the O-glycan dense region is proteolytically resistant to commensal bacteria, mammalian mucin degradation in general is thought to be initiated with glycan removal (Winchester, 2005; Madsen et al., 2015). Consequently, the entire peptide backbone is then accessible to a range of enzymes and the degradation can be completed. However, it is noteworthy that O-glycoproteases expressed by pathogens typically demonstrate increased activity and do not necessitate prior removal of O-glycans for hydrolysis (Taleb et al., 2022). Besides enzymatic breakdown, mucin degradation can also occur through shedding from the cell surface to luminal spaces (Kim et al., 1999). While the latest findings have provided new insights into novel enzymatic mechanisms targeting non-salivary mucin, a comprehensive characterization of MUC5B degradation is yet to be realized. Conversely, non-salivary O-glycans are typically less complex and are considerably shorter than the extensive MUC5B O-glycans (Thomsson et al., 2002; Jin et al., 2017). O-glycoproteases, O-glycanases, and mucinases have not yet been identified in oral bacteria, suggesting that these bacteria are potentially employing an entirely distinct enzymatic approach for the degradation process.

The characterization of a recently discovered MUC5B degrading protease, MdpL from *Limosilactobacillus fermentum*, revealed a new type of enzyme that facilitates MUC5B hydrolysis (Leo et al., 2023). MdpL hydrolyzed fragmented MUC5B and also preferred substrates with a high O-glycan content. The enzyme displayed O-glycan independency, although it had a higher activity on O-glycosylated (and de-glycosylated) proteins compared to non-glycosylated proteins such as albumin and IgG, hinting that it may prefer hydrolysis of targets with amino acid sequences promoting O-glycosylation rather than the glycan itself. Furthermore, it had a high sequence homology to other oral bacterial genera, which suggests an important and conserved function in MUC5B-rich environments. However, *L. fermentum* quantitively constitutes a very low percentage in a typical biofilm, and therefore it is crucial to examine predominant commensals to gain a comprehension of the biological significance of these conserved enzymes. In this paper, we have studied a protease from *S. oralis* sharing a high sequence homology with MdpL. The enzyme was characterized in regard to its physicochemical properties, substrate - and amino acid specificity, cellular localization, and hydrolytic interactions with MUC5B. Because of its mucin-degrading function, the protein will be denoted as MdpS (Mucin Degrading Protease from *Streptococcus oralis*).

## Materials and methods

### Sequence homology of bacterial enzymes

**Figure 1:**
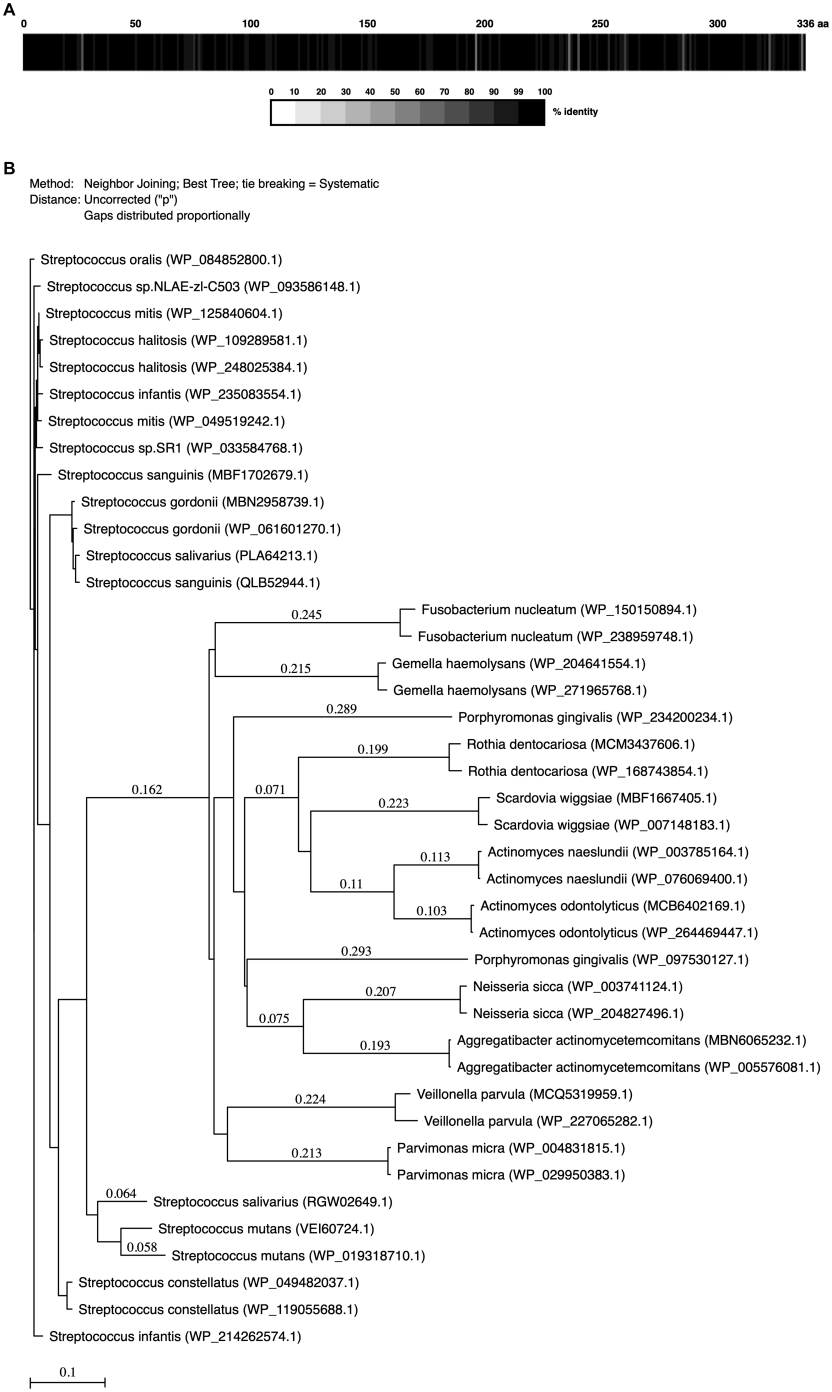
*Streptococcus oralis* MdpS alignment and degree of protein sequence conservation with oral bacteria. **(A)** The box displays the percentage (%) of the conserved amino acid identity of all sequenced proteomes of *Streptococcus oralis* compared to one selected *S. oralis* strain (GenBank: WP_084852800.1). The color key below shows the correlation between the greyscale and the level of identity. **(B)** Phylogenetic tree of MdpS and related proteins from oral species. The number above the branch points denotes the confidence level of the relationship of the paired sequences determined by bootstrap statistical analysis. The tree is drawn to scale.

Sequence homologies of MdpS (GenBank accession number: WP_084852800.1) from all 113 *S. oralis* strains annotated on GenBank, as well as duplicate strains of a number of selected oral species were calculated using the Protein Alignment and ClustalW function in MacVector version 18.5.1 (23; MacVector Inc., United States). The selected oral species’ respective GenBank accession numbers are included in Figure 1B. A phylogenetic tree was constructed, using MacVector based on neighbor-joining and bootstrap analysis. The values on each branch represent the estimated confidence limit (%) for the position of the branch.

### Recombinant expression and purification of MdpS

Codon-optimized gene constructs representing the whole MdpS protein (GenBank accession number: WP_084852800.1) containing no predicted signal peptide were inserted into a pET21(a) plasmid (Genscript, United States). WP_084852800.1 was selected because of its high identity, among available *S. oralis* genomes, to MdpL from *L. fermentum* (KRN17993.1). It was recombinantly expressed in *E. coli* BL21(DE3) STAR cells as a fusion protein with a C-terminal Gly-Ser-Gly (GSG) linker and 6xHis-tag. The FASTA sequence of MdpS can be found in Supplementary Figure 1. Bacteria were cultured (37°C, 220 rpm) in Luria Broth until OD_600_ reached 0.6 and the cells were induced with 1 mM IPTG. Protein expression was continued for 5 h (37°C, 220 rpm), before collecting and freezing cells (−20°C). The cells were thawed and resolved in a His binding buffer (20 mM NaP, 500 mM NaCl, 20 mM imidazole, pH 7.4), before intracellular proteins were released using sonication (6 × 5 min, 70% efficiency, 50% power) with SONOPULS homogenizer (Bandelin GmbH, Germany). After centrifugation, the supernatant was affinity purified by adding the solution to pre-equilibrated His GraviTrap columns (Cytiva, United States) followed by washing using additional His binding buffer, to remove non-His-tagged proteins. MdpS was eluted from the column using His elution buffer (20 mM NaP, 500 mM NaCl, 500 mM imidazole, pH 7.4) before being re-buffered to a TBS buffer (50 mM Tris, 150 mM NaCl, pH 7.6) on a PD-10 column (Cytiva). The protein concentration was determined using Nanodrop Spectrophotometer-ND1000 (Thermo Fisher Scientific, United States), and the purity was estimated through SDS-PAGE.

### Physicochemical characterization of MdpS

The conditions allowing optimal MdpS activity were assessed. The effect of temperature (4, 25, 34, 37, 40, 50°C), cations/EDTA (2 mM CaCl_2_, MgCl_2_, ZnCl_2_, EDTA), NaCl (0–1000 mM) and reducing agents [L-cys and DTT (2–40 mM)] on MdpS activity toward Etanercept (Pfizer Inc., United States) was determined using SDS-PAGE with densitometric quantification using Image Lab software (Version 6.0, Bio-Rad laboratories, United States). The IgG1 fusion antibody Etanercept was selected because of its glycan pattern similarities to MUC5B. Furthermore, enzyme kinetics are substrate-dependent and consequently, it is more relevant to use Etanercept than standard soluble substrates to generate more biologically applicable data. The pH optimum was determined using 100 mM sodium acetate buffer, pH 5.0–5.5, 50 mM Bis-Tris buffer, pH 6.0–6.5, and 50 mM Tris buffer, pH 7.0–9.0, followed by densitometric quantification. The amount of hydrolyzed substrate was calculated by dividing the separate measured activity values with the unhydrolyzed substrate control. In all assays, MdpS was incubated with Etanercept (16 h, 37°C) in technical triplicates.

### Assessing MdpS proteolytic inhibition

Potential inhibition of the proteolytic activity of MdpS was studied with a panel of protease inhibitors: AEBSF, ALLN, antipain, bestatin, chymostatin, E64, leupeptin, pepstatin, phosphoramidon, and PMSF (G-Biosciences, Geno Technology Inc., United States) following the manufacturer’s instruction. EDTA-Na_2_ was excluded from the panel since its cation-chelating effect was shown in the physicochemical tests described above. MdpS inhibition was analyzed by gel electrophoresis using Etanercept as the substrate, followed by densitometric quantification.

### Substrate specific activity of MdpS

The substrate-specific activity was measured with densitometric quantification from reduced SDS-PAGE, after MdpS incubation with IgG (1,2,3,4), IgE, IgM, CTLA-4-IgG1 (Abatacept), TNFR-IgG1 (Etanercept), Lactoferrin, Albumin and Fetuin in 0.1 M Tris, pH 7.5, with 2 mM Ca^2+^ (18 h, 37°C). Humanized IgG1 Trastuzumab (Roche, Switzerland), human IgG2 Panitumumab (Amgen, United States), human plasma IgG3 myeloma (Merck), human IgG4 Nivolumab (Bristol-Myers Squibb, United States), native pIgA purified from plasma (Calbiochem, United States), human IgE myeloma (Merck), human serum IgM (Merck), fusion protein Abatacept (Bristol-Myers Squibb), fusion protein Etanercept (Pfizer Inc.), fetal calf serum Fetuin (NEB, United States), human serum Albumin (Merck), and bovine Lactoferrin (Merck) were used.

### Degradation of MUC5B analyzed by SDS-PAGE

Human MUC5B purified from whole saliva as previously described by Wickström and Svensäter (2008) was incubated with MdpS (24 and 48 h, 37°C) and 2 mM Ca^2+^ in 50 mM Tris [pH 7.5]. The reactions were quenched in the freezer before they were denatured, reduced, and heated in 4 M urea, 100 mM DTT, and LDS (1 h, 95°C). An alkylation step using 45 mM iodoacetamide (1 h, 25°C) prevented the re-formation of disulfide bonds. The samples were then loaded onto a 3–8% Tris-Acetate gel (Thermo Scientific) in a two-step electrophoresis run; a 10 V constant run (20 min, 4°C), followed by a 200 V constant run (65 min, 25°C).

### Periodic acid-Schiff, Coomassie, and silver staining of MUC5B

Following the gel electrophoresis described above, the gel was fixed and then incubated in 1% periodic acid (Sigma), 3% acetic acid (30 min, 25°C). Sodium metabisulfite (Sigma) was used to wash the gel prior to the reaction with the Schiff’s reagent (Sigma; 1 h, 25°C). The reaction was quenched in sodium metabisulfite, after which the gel was analyzed using the Image Lab software (Bio-Rad laboratories). Instead of staining with PAS, a second gel was run as described above, but thereafter double stained with Coomasie and Silver (Sigma), followed by image analysis immediately after band development.

### MUC5B and salivary IgA sample preparation for NanoLC–MS analysis

Allowing more detailed hydrolytic data of the MdpS activity toward MUC5B from whole saliva, 100 μg enzyme was employed in a reaction with ~50 μg MUC5B in 2 mM Ca^2+^ in 50 mM Tris, pH 7.5 (48 h, 37°C). A MUC5B control sample without the enzyme was also included. MUC5B (25 μg) with or without MdpS was reduced in 25 mM DTT (30 min, 60°C), followed by alkylation in 62.5 mM iodoacetamide (20 min, 25°C in the dark). The samples were acidified and desalted on C18 columns (Pierce Peptide desalting spin columns, Thermo Fisher Scientific) according to the manufacturer’s instructions, followed by vacuum centrifugation to dryness.

Salivary IgA was inadvertently included in the MUC5B preparation from whole saliva, and consequently, the amount could not be determined meaning the sample preparation was not optimized for IgA.

### NanoLC–MS analysis of MUC5B and salivary IgA hydrolysis

NanoLC–MS/MS was performed on an Orbitrap Exploris 480 mass spectrometer interfaced with an Easy-nLC1200 liquid chromatography system (both Thermo Fisher Scientific). Peptides were trapped on an Acclaim Pepmap 100 C18 trap column (100 μm × 2 cm, particle size 5 μm, Thermo Fischer Scientific) and separated on an in-house packed analytical column (75 μm × 35 cm, particle size 3 μm, Reprosil-Pur C18; Dr. Maisch, Germany) using 90-min runs with a gradient from 5 to 35% ACN in 0.2% formic acid at a flow of 300 nL/min. Each preparation was analyzed using MS^1^ scan settings, m*/z* 350–2000, at a resolution of 120 K. MS^2^ analysis was performed in data-dependent mode at a resolution of 30 K, using a cycle time of 2 s. The most abundant precursors with charges 2–6 were selected for fragmentation using HCD at collision energy settings of 30. The isolation window was set to 1.2 *m/z and* the dynamic exclusion and intensity threshold (IT) was set to 10 ppm for 12 (IT = 50,000) or 30 s (IT = 10,000).

### Proteomic data analysis

The raw files from the NanoLC–MS run were searched using Proteome Discoverer version 2.4 (Thermo Fisher Scientific). The data were matched against the human *Swissprot* database (May 2022) using Sequest as a search engine. The precursor mass tolerance was set to 5 ppm and the fragment mass tolerance to 0.05 Da. Peptides consisting of at least 5 amino acid residues formed by unspecified cleavage were searched for. The target false discovery rate was set to 1%. The spectra were manually inspected to include both the b- and y-ion series to verify that the start and end cleavage residues were correctly assigned. Variable modifications for methionine oxidation and fixed for carbamidomethylation were selected.

A search for glycopeptides was conducted on the raw files using Byonics (Protein Metrics) against human MUC5B and S-IgA. The precursor mass tolerance was set to 5 ppm and fragment mass tolerance to 20 ppm. The search criteria were limited to two modifications per peptide and searched against 52 common *N*-glycans and 9 most common *O*-glycans. Spectra with scores above 300 were included and validated manually. Unspecified cleavages were searched for. In addition, cleavages on the N-terminal side of Ser/Thr and on the C-terminal side of Ser/Thr were used in separate Byonic searches. These Ser/Thr searches were conducted with up to four allowed missed cleavages. The mass spectrometry proteomics data have been deposited to the ProteomeXchange Consortium via the PRIDE (Perez-Riverol et al., 2022) partner repository with the dataset identifier PXD046810.

### LC–MS/MS analysis of Etanercept hydrolysis

Native or deglycosylated Etanercept (Pfizer Inc.) was incubated with or without MdpS in 2 mM Ca^2+^ in 50 mM Tris, pH 7.5, prior to quenching the reaction with 2 mM Zn^2+^. The N- and O-glycans were removed before or after MdpS incubation by SialEXO (Genovis), OglyZOR (Genovis), GalNAcEXO (Genovis), and PNGaseF (Genovis; 4 h, 37°C) to evaluate MdpS glycan dependence and to reduce the peptide mass variation, thus simplifying the analysis. Fragments were separated with LC (Agilent Technologies 1290 Infinity; Agilent, United States) using a C18 column (2.1 × 150 mm, particle size 1.7 μm, ACQUITY Premier CSH C18; Waters, United States) with 60 min runs using a gradient from 2 to 50% ACN in 0.1% formic acid at a flow of 0.2 mL/min and a column temperature of 50°C. The peptide fragments were analyzed by MS/MS using an Electronspray Ion Source Time-Of-Flight (ESI-TOF) mass spectrometer (Bruker Impact II). Data were acquired using positive ion mode, and mass spectrometry analysis was conducted with the ESI source temperature set at 200°C and a source voltage of 4.5 kV. The gas pressure maintained during the analysis was 1.6 Bar, with a consistent gas flow rate of 8.0 milliliters per minute. MS-spectra were acquired at a rate of 2 Hz, with precursor selection confined within the range of 150 to 3,000 m/z. For ion manipulation, the quadrupole ion energy was set at 5.0 electronvolts (eV), while the collision cell energy was adjusted to 7.0 eV. Isolation and subsequent fragmentation occurred within the mass range of 150 to 1,300, employing energies ranging from 23 to 65 eV for efficient fragmentation of selected ions. MdpS hydrolysis sites were mapped using the Bruker Compass Data Analysis 5.2 and BioPharma Compass 4.0 software. The parameters used for the peptide search included a non-specific hydrolysis with allowance for 20 missed cleavages, employing a peptide tolerance of 10 ppm, and an MS/MS tolerance of 0.05 Da. Peptide hits meeting the criteria for acceptance were required to attain a score equal to or greater than 30.0, along with a fragmentation coverage, and an intensity coverage exceeding 5 and 0.1%, respectively. The mass spectrometry proteomics data have been deposited to the ProteomeXchange Consortium via the PRIDE (Perez-Riverol et al., 2022) partner repository with the dataset identifier PXD046954.

### Amino acid position weight matrices

Four sequence logos displaying the most probable amino acid ±3 positions from the MdpS hydrolytic site were created using Graphpad Prism 9 (Graphpad Software Inc., United States). The hydrolytic site data from 445, 53, and 80, respectively, high-score peptides generated from the hydrolysis of MUC5B, Etanercept, and S-IgA were uploaded to Weblogo (Version 3.0, University of Berkeley). The data output specified the quantity of the amino acids at each specific position (1–6) and the numbers were entered into Excel and a probability percentage (%) was calculated.

### LC–MS analysis of MdpS autoproteolysis

MdpS autoproteolysis was allowed in a reaction with 2 mM Ca^2+^ in 50 mM Tris, pH 7.5, using different incubation times, *viz.* 0, 2, 4, 8, 16, 24 h (37°C). The reactions were immediately quenched with 2 mM Zn^2+^ and kept in the freezer (18 h, −20°C) before denaturing and reducing using 4 M Guanidine Hydrochloride and 0.1 M DTT (1 h, 37°C). Protein fragments were separated with LC (Agilent Technologies 1,290 Infinity; Agilent) using a C4 column (2.1 × 100 mm, particle size 1.7 μm, ACQUITY Premier Protein BEH C4; Waters, United States) with 42-min runs using a two-step gradient from 10 to 25% ACN and 25 to 40% ACN in 0.1% formic acid at a flow of 0.2 mL/min and a column temperature of 60°C. The fragments were analyzed by MS^1^ using a ESI-TOF mass spectrometer (Bruker Impact II). Data were acquired using positive ion mode, and mass spectrometry analysis was conducted with the ESI source temperature set at 220°C and a source voltage of 4.5 kV. The gas pressure maintained during the analysis was 1.8 Bar, with a consistent gas flow rate of 8.0 mL per minute. MS-spectra were acquired at a rate of 2 Hz, with precursor selection confined within the range of 300 to 3,000 m/z. MdpS hydrolysis sites were mapped using the Bruker Compass Data Analysis 5.2 software, in which the data was first deconvoluted into the correct compound masses using the maximum entropy (MaxEnt) algorithm. The mass spectrometry proteomics data have been deposited to the ProteomeXchange Consortium via the PRIDE (Perez-Riverol et al., 2022) partner repository with the dataset identifier PXD046954.

### Cell localization analysis of MdpS

The cell localization of MdpS was evaluated by analyzing the content in three different samples: papain-digested cells (surface associated: cell wall-attached), sonicated cells (lysate: intracellular and cell wall-attached), and the bacterial supernatant (extracellular). The *S. oralis* ATCC 9811 strain was cultivated in Brain Heart Infusion (BHI) broth (17 h, 37°C) after which the cells and supernatant were separated and stored in the freezer (−20°C). One cell fraction was dissolved in 0.1 M Tris, pH 7.6, (5% SDS) before being sonicated for release of intracellular proteins (6 × 5 min with an equally long pause between each step, 70% efficiency, kept on ice) after which the supernatant was collected. A second cell fraction was incubated with 5 μg papain (Sigma) and L-cys (1 h, 37°C), prior to inhibiting further hydrolysis with leupeptin (Sigma) and collecting the supernatant. The *S. oralis* supernatant was concentrated and re-buffered in PBS before being analyzed. The three samples were incubated with trypsin (Promega, United States), separated with a EvoSep One LC system (nano-UHPLC; Evosep, Denmark), and MS/MS data were acquired via online connection using a Q Exactive HF-X Quadrupole-Orbitrap mass spectrometer (Thermo Scientific). Approximately 400 ng peptides were injected using the Whisper 20 SPD LC method. The LC column used was an in-house packed SelfPack NanoLC column with emitter tip and frit (MS Wil 360 μm OD × 75 μm ID × 15 cm L, 15 μm tip ID) with Reprosil-Pur-Basic-C18, 1.9 μm packing material (Dr. Maisch). The MS was operating in data-dependent acquisition mode using a 58 min top 20 method. MS1 target resolution was 120,000 and MS2 target resolution was 15,000. Normalized collision energy 27 was used for MS/MS fragmentation. The LC–MS/MS data were searched in MaxQuant (version 1.6.17.0) against a database consisting of the *S. oralis* SK304 Uniprot sequences, supplemented with WP_139689690 tRNA (adenosine(37)-N6)-threonylcarbomoyltransferase complex transferase subunit TsaD protein sequence. The mass spectrometry proteomics data have been deposited to the ProteomeXchange Consortium via the PRIDE (Perez-Riverol et al., 2022) partner repository with the dataset identifier PXD046954.

### SDS-PAGE analysis of Etanercept hydrolysis in *Streptococcus oralis* supernatant

The potential presence of MdpS in the extracellular fraction of *S. oralis* was assessed using SDS-PAGE. The *S. oralis* supernatant was prepared and concentrated in PBS as described above. The supernatant, together with four recombinant MdpS replicates, was then subjected to Etanercept hydrolysis in a reaction with 2 mM Ca^2+^ in 50 mM Tris, pH 7.5 (18 h, 37°C). The activity was evaluated using SDS-PAGE, at which the hydrolytic pattern of all samples was compared to identify possible similarities.

## Results

### MdpS-homologues is highly conserved among early biofilm colonizing streptococci

MdpS conservation across common oral bacteria was investigated using various bioinformatic methods (Figure 1). Analyzing all sequenced *S. oralis* strains from public search domains revealed a very low incidence of changed amino acids of the MdpS protein sequence, suggesting a high species conservation (Figure 1A). Single amino acid polymorphisms were detected for at least 10 strains at 16 different positions spread throughout the sequence. These changes entailed major functional alterations in 10 of the amino acid positions, which probably do not affect the biological properties in general. Apart from the *S. oralis* species, MdpS had the highest homology with other early biofilm colonizers in the mitis group within the *Streptococcus* genus (Figure 1B). Other oral streptococci with high MdpS correspondence were divided into three different groups, where *Streptococcus constellatus* had the highest, *Streptococcus gordonii* had a moderate, and *Streptococcus mutans* had the lowest homology. The intermediate and late biofilm colonizers *Parvimonas micra*, *Gemella haemolysans*, *Veillonella parvula*, and *Fusobacterium nucleatum* were far more distantly related and the disease-associated *Porphyromonas gingivalis* and *Aggregatibacter actinomycetemcomitans* seemed to lack an MdpS-like enzyme. Interestingly, so did the early colonizers *Actinomyces naeslundii*, *Neisseria sicca*, and *Rothia dentocariosa*.

### MdpS is a serine protease with narrow physicochemical preferences revealing the environmental niche of *Streptococcus oralis*

**Figure 2:**
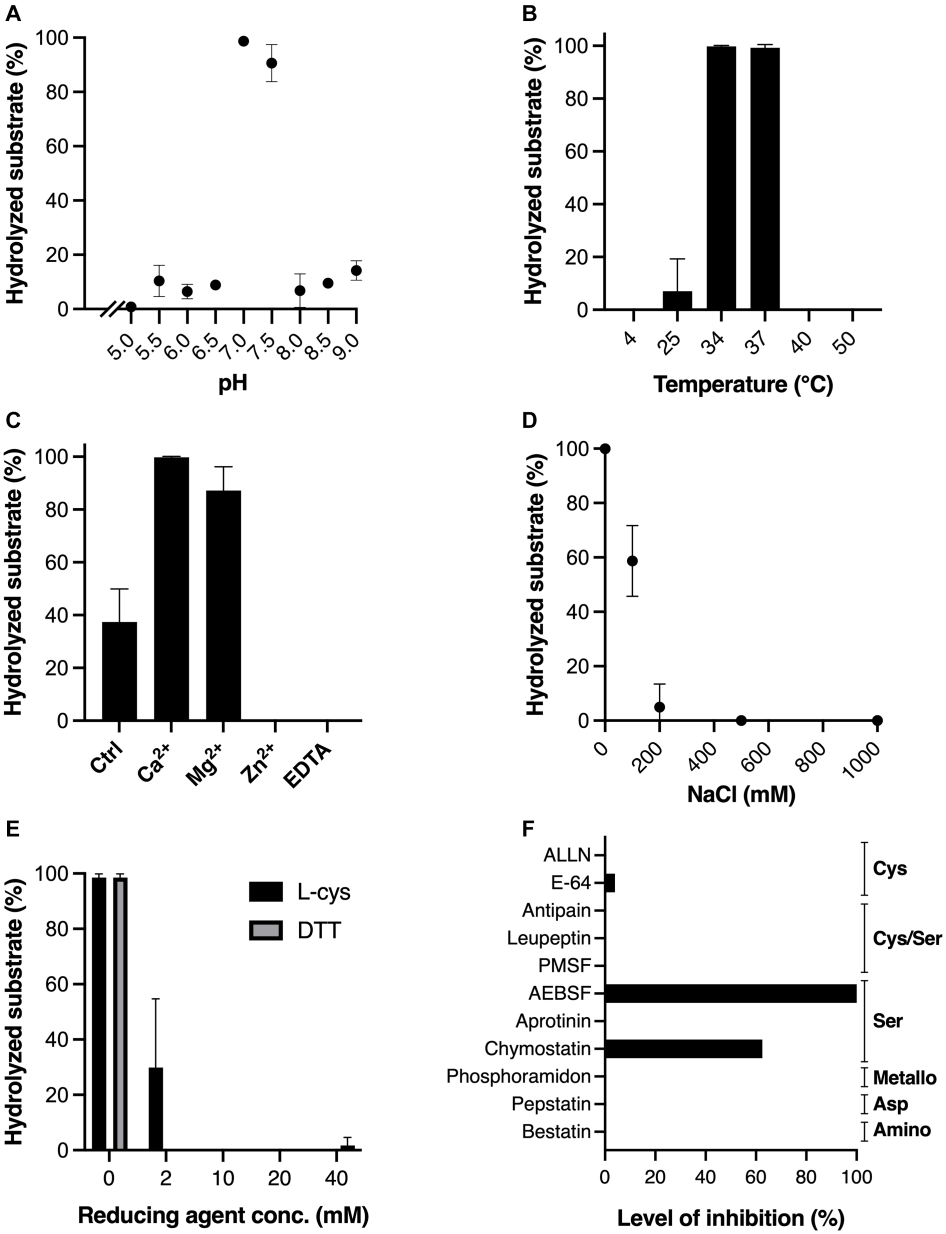
Impact of different physicochemical conditions and protease inhibitors on MdpS activity. Effect of **(A)** pH, in the interval 5.0–9.0, **(B)** a wide range of temperatures (4–50°C), **(C)** 2 mM Ca^2+^, Mg^2+^, Zn^2+^, and EDTA, **(D)** increasing NaCl concentrations (0–1,000 mM), **(E)** the reducing agents L-cys and DTT at increasing concentrations (0–40 mM), **(F)** common protease inhibitors, where the inhibition level is displayed as percentage (%) in relation to baseline MdpS activity. Common protease families inhibited by the protease inhibitors are noted to the right of the graph. All assays were conducted as technical triplicates and relied on SDS-PAGE and densitometric quantification. The amount of hydrolyzed substrate in A-E is the calculated percentage of digested substrate. The baseline control in C-F had hydrolyzed 37.4, 100, 98.5, and 100% substrate, respectively. Data in A-E is plotted as mean values ± SD.

The physicochemical properties of MdpS were determined with Etanercept as the selected substrate, where the level of hydrolysis was analyzed using gel electrophoresis with densitometric quantification (Figure 2). The pH range that MdpS could accept was very small, from 6.5 to 7.0 only (Figure 2A), and its optimal temperatures were at or a bit below normal body temperature (Figure 2B). The divalent cations Ca^2+^, and Mg^2+^ clearly increased the activity (Figure 2C), whereas sodium chloride concentrations above 100 mM had a strong negative effect on the activity (Figure 2D). The activity of MdpS was markedly negatively affected by both reducing agents tested (Figure 2E). The protease inhibition test revealed a partial or complete lack of activity caused solely by the group of serine protease inhibitors (Figure 2F). Representative gels for each test can be found in Supplementary Figures 2–6. The pH buffers used were not corrected for ionic strength which could affect the shape of the curve and give slightly unfitting values. The biological conclusions will thus remain unaffected by such a modification.

### MdpS demonstrates substrate preference for O-glycosylated proteins

**Figure 3:**
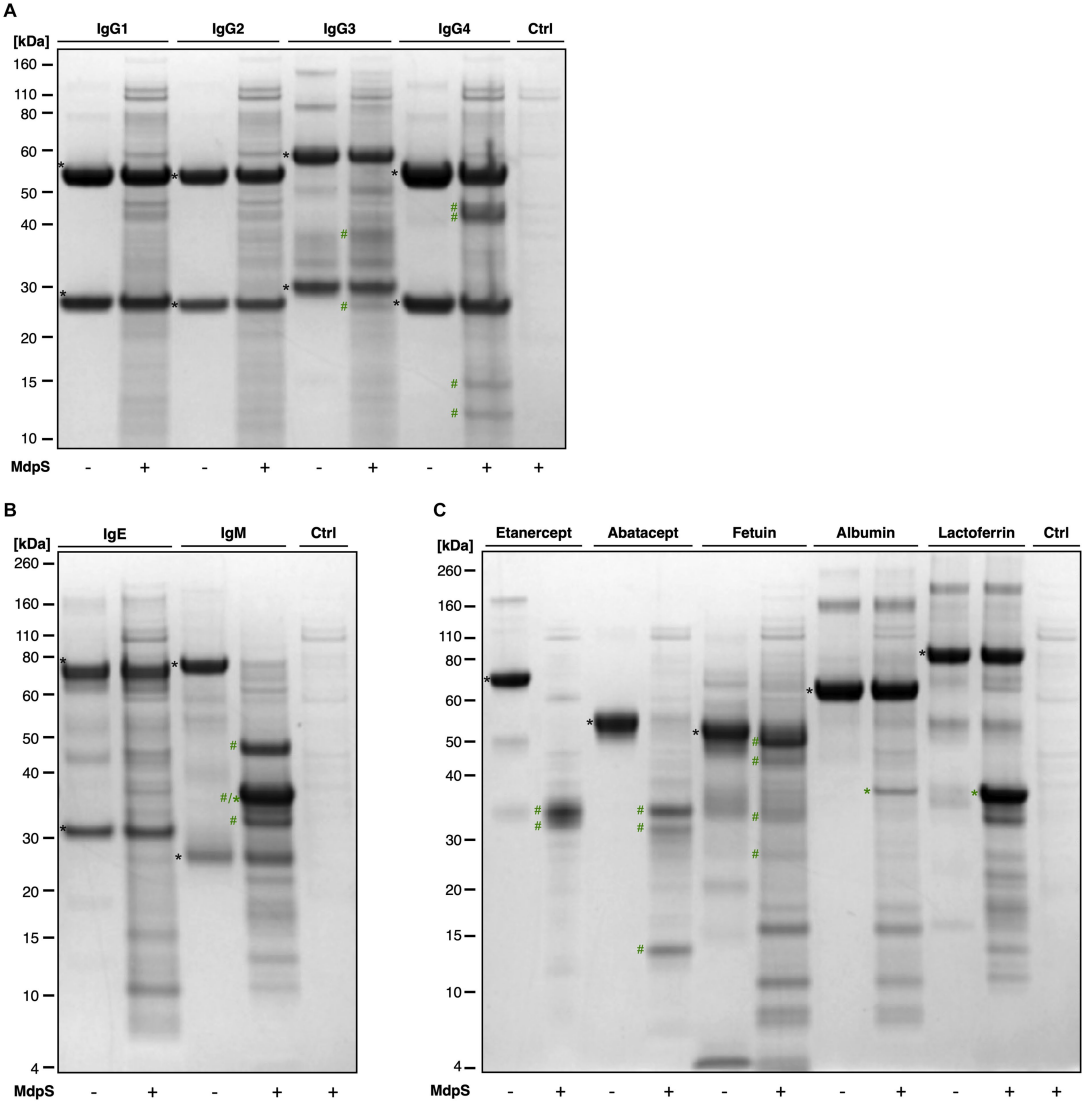
MdpS substrate specificity assessment: **(A)** human IgG subclasses, **(B)** human IgE and IgM, **(C)** O-glycoproteins and non-glycosylated substrates. All protein substrates, encompassing IgG1-4, IgE, IgM, Etanercept, Abatacept, bovine fetuin, human serum albumin, and lactoferrin were incubated with MdpS (18 h, 37°C) and were then analyzed using SDS-PAGE under reducing conditions. Intact substrates (black asterisk), intact MdpS (green asterisk), and MdpS-generated fragments (green hashtag).

The substrate specificity of MdpS was evaluated by testing the activity toward different Immunoglobulins, O-glycoproteins, and non-glycosylated proteins (Figure 3). Neither IgG1, IgG2, nor IgE were significantly hydrolyzed by MdpS, however, minor hydrolysis was seen on IgG3 and IgG4 (Figures 3A,B). In contrast, IgM was completely degraded (Figure 3B), which suggests a possible immunomodulatory effect of MdpS. Interestingly, all tested O-glycoproteins were degraded to a high extent, whereas the non-glycosylated substrates albumin and lactoferrin remained intact after incubation (Figure 3C). LC–MS^2^ analysis of intact and deglycosylated Etanercept following MdpS incubation revealed pronounced hydrolysis N- and C-terminally of the O-glycan-rich region, in contrast to the control (Supplementary Figures 7A–C). Furthermore, the data displays highly similar peptide maps when comparing the hydrolysis of non- and deglycosylated Etanercept, suggesting independence from O-glycan features.

### MdpS-mediated hydrolysis of salivary IgA1 and IgA2

To further evaluate if MdpS could have any potential immunomodulatory effect, the activity on salivary IgA was assessed using NanoLC–MS and Proteomic Data Analysis (Supplementary Figure 8). After being subjected to MdpS incubation, a significant number, *i.e.*, 54, unique peptides derived from the kappa, lambda, J, and IgA1/2 CH1-3 chain of salivary IgA were identified. Interestingly, the data indicated that MdpS hydrolysis was ineffective in the O-glycosylated region of IgA1. However, apart from this, the IgA1 digestion with a 57% sequence coverage, is more comprehensive compared to the constant heavy chains of IgA2, which had a lower, *i.e.*, 41%, sequence coverage. Ultimately, this emphasizes the preference for protein substrates with O-glycosylation, without hydrolyzing in the O-glycan-rich regions as presented in Figure 3.

### MUC5B is extensively degraded in the N- and C-terminal domains

**Figure 4:**
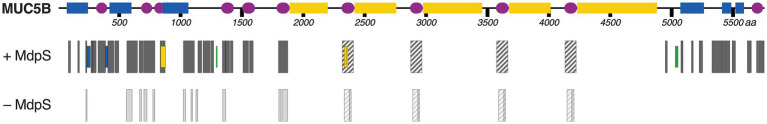
Schematic overview of MUC5B degradation by MdpS. The different colors in the MUC5B illustration depicts important protein domains; Blue – von Willebrand factor-like dimerization domains containing N-glycosylation sites, Purple – cysteine-rich domains with a high percentage of disulfide bridges, Yellow – Pro/Ser/Thr-rich domains with a vast number of O-glycosylation sites. The peptide quantity profoundly increased when MdpS was incubated with MUC5B, as compared to the control sample. The filled black and gray boxes are the identified MUC5B peptides. Boxes with a diagonal stripe included peptides that have the same primary sequence at four separate sites, and their exact positions could not be determined. Blue, yellow, and green boxes are N-, O-, or N/O- glycopeptides respectively, which could only be found in the MdpS sample.

Two approaches were employed to assess the degradation of MUC5B. In the initial analysis, MUC5B degradation was evaluated via SDS-PAGE after incubation with MdpS for 24 and 48 h (Supplementary Figure 9). Silver-staining, targeting the peptide backbone, reveiled a downward band migration of high molecular weight bands following a 24-h incubation with MdpS and a complete absence of observable bands after 48 h of incubation. Similar results was seen using PAS-staining, which targets the mucin glycans specifically, unveiling a reduction in the diffuse high molecular weight bands (~1 MDa) following a 48-h treatment with MdpS. The diminished intensity observed at the top of the well compared to the control sample indicated a decreased presence of intact MUC5B, further corroborating the degradation of MUC5B. In the second experiment, untreated MUC5B from whole saliva was incubated with MdpS for 48 h and was thereafter prepared for NanoLC-MS analysis (Figure 4; Supplementary Figure 10). Interestingly, a total of 423 unique peptides and 22 N- and O-glycopeptides, all originating from MdpS hydrolysis, were identified, resulting in a cumulative sequence coverage of 27%. The peptides cover most domains within MUC5B, but notably, they are absent from the regions rich in O-glycans. With the used Byonic searches described in the method section, it was also tested if the protease could hydrolyze similarly to the previously described O-glycoproteases, in direct proximity to an O-glycosylated Ser/Thr. However, no additional O-glycopeptides could be identified with this approach.

### MdpS employ hydrolytic amino acid discrepancy depending on the substrate glycosylation

**Figure 5:**
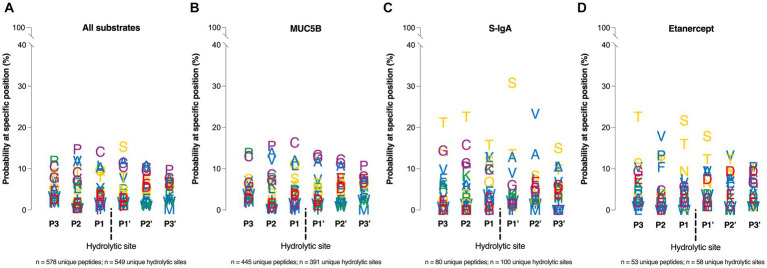
Amino acid specificity analyzed by NanoLC–MS, and LC–MS/MS. Four Position Weight Matrices was constructed based on MdpS hydrolysis of **(A)** All substrates summarized **(B)** MUC5B, **(C)** S-IgA, **(D)** and Etanercept. The y-axis position of each letter is based on the probability of a particular amino acid on a specific position ±3 residues from the hydrolytic site (dashed line). The matrices were based on the 445, 53, and 80 identified unique peptides from the MUC5B, Etanercept, and S-IgA hydrolyses, respectively. Amino acids are given a color based on the side chain; Green – positive charge, Red – negative charge, Yellow – uncharged, Purple – special cases, Blue – hydrophobic.

Assessing the amino acid specificity of MdpS, heavily O-glycosylated proteins were chosen as the most endogenic substrates. The MdpS-derived peptides consisting of three amino acids N- and C-terminally of each hydrolytic site were categorized and summarized in one, but also divided into three separate lists corresponding to the substrates: MUC5B, S-IgA, or Etanercept. Combining the data from all substrate hydrolyses, MdpS seems to a minor preference for serine at position P1’, and an probability increase of proline and cysteine at position P2 and P1, respectively (Figure 5A). The MUC5B profile revealed a preference for cysteine, proline, glycine, and alanine around the hydrolytic site (Figure 5B). Out of the 892 peptides, approximately 10% had a cysteine present in position P3-P3’, but cysteine was not clearly overrepresented in any position. In contrast, in the S-IgA profile, a few amino acids stand out from the rest, notably with a probability exceeding 30% for serine at the P1’ position (Figure 5C). Despite MdpS not hydrolyzing the O-glycan rich region of IgA1, it does exhibit a preference for Ser/Thr and to some extent also Val/Ala. Similarly, Etanercept is subject to hydrolysis near Ser/Thr residues, with a notable 35–40% probability of encountering either of these amino acids in the P1 or P1’ position (Figure 5D).

### Extensive initiation time necessary in complete autoproteolytic degradation of MdpS

The autoproteolytic ability and specificity of MdpS were evaluated using LC-MS analysis (Supplementary Figure 11, Table 1). The data revealed a gradual initial phase, during which 90% of intact MdpS persisted after 4 hours. Subsequently, a more pronounced hydrolysis stage commenced around the eighth hour, resulting in near-complete degradation after 16 h. The identified MdpS fragment preceding the intense phase displays a hydrolysis in the C-terminus after amino acid position 307, between Met/Ile/Ala and Tyr/Ala/Ser (Supplementary Figure 12, Table 1). Assessing the enzymatic hydrolytic potential, we examined seven distinct ratios of MdpS to Etanercept, spanning from 1:80 to 2:1 (Supplementary Figure 13). The data revealed that achieving complete protein degradation high enzyme quantities were necessary.

### MdpS can be presented extracellularly by *Streptococcus oralis*

The cellular localization of MdpS was explored by fractionating a *S. oralis* culture into cellular and secreted fractions (Supplementary Figure 14). The cellular fraction was treated using sonication or papain, resulting in lysate and surface-associated fractions, respectively. These two exclusively displayed XIC intensity from MdpS peptides, in contrast to the extracellular fraction which gave a null result (Supplementary Figure 14A). The total intensity was almost exclusively represented by the lysate sample, suggesting most enzyme is found intracellularly. Similarly to the intensity results, unique MdpS peptides were identified in the same fractions (Supplementary Figure 14B). However, due to the autoproteolytic nature of MdpS, the results could be contradictory. Further supporting the idea of an extracellular localization of MdpS, MdpS-like activity could be detected in the crude supernatant of *S. oralis* (Supplementary Figure 15).

## Discussion

**Figure 6:**
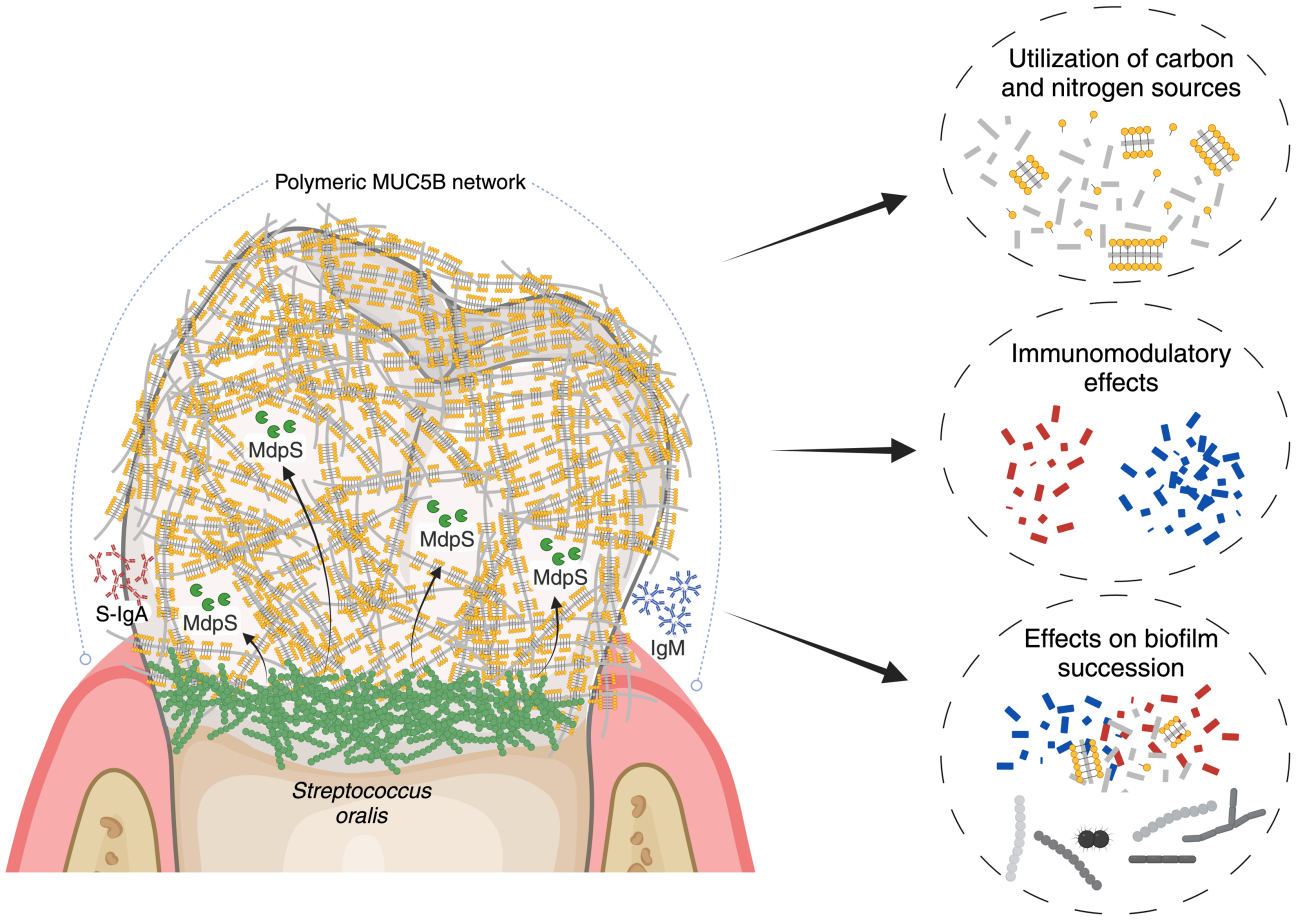
Illustrative overview of MdpS’ potential role in the oral environment. The early colonizer *S. oralis* adheres to the salivary pellicle, which contains the predominant glycoprotein MUC5B, and is also detected by the humoral immune system. In an early colonization phase, it is suggested that MdpS, which is potentially presented extracellularly, degrades MUC5B, S-IgA and IgM. The concrete biological purpose of the enzyme was not investigated, but it could have central implications in nutrition, immunomodulation, and/or biofilm succession. The figure was created with BioRender.com.

The role of *S. oralis* as a significant commensal bacterium in the initial biofilm formation process has been extensively studied. Existing knowledge about how these bacteria interact with MUC5B includes their utilization of glycan-specific adhesins for binding and a range of glycosidases to hydrolyze glycans for nutrient sequestration. Nevertheless, the molecular mechanisms responsible for degrading the peptide backbone of MUC5B have not been thoroughly investigated. In a recent study, the discovery of MdpL, a protease from *L. fermentum*, drew attention due to its degrading activity on MUC5B and the high sequence homology to numerous oral commensal bacteria (Leo et al., 2023). This in-depth study introduces MdpS, an O-glycan-independent protease from *S. oralis* possessing highly specific physicochemical properties, and is suggested to degrade MUC5B, IgA and IgM after putative extracellular presentation (Figure 6).

The cellular localization analysis indicated that MdpS, similarly to MdpL (Leo et al., 2023), is presented extracellularly (Supplementary Figure 14). An absent intensity signal from the secreted fraction was observed, but it is important to acknowledge that the autoproteolytic nature of MdpS could introduce some complexity and ambiguity in the results. The finding when assessing activity toward Etanercept in the supernatant of *S. oralis*, which resulted in a MdpS-like hydrolysis, further strengthens the extracellular presentation of MdpS (Supplementary Figure 15). Interestingly, proteomics studies have unveiled instances where proteins initially annotated as cytoplasmic can be identified extracellularly due to non-canonical secretion pathways (Siciliano et al., 2019). Moreover, an extracellular location would be particularly advantageous for the enzyme, considering its proximity to MUC5B. Further investigations, including immunolocalization and proteomic approaches, may be warranted to clarify these findings and provide a more comprehensive understanding of the biology of MdpS.

The MdpS sequence homology study suggests a unique presence of MdpS-like enzymes within Streptococci. The results presented in Figure 1 aligns well with the phylogenetic tree of the *Streptococcus* genus, encompassing its eight established groups as previously outlined (Richards et al., 2014). Notably, the highest homology is observed within the mitis group, while the anginosus group exhibits a closer relationship with MdpS from *S. oralis* than with the sanguinis group. The salivarius group appears somewhat more distantly related, and it is important to also note that among all the oral streptococci analyzed in this study, the mutans group demonstrates the lowest degree of similarity to MdpS. Whether this lower homology suggests a different hydrolytic function needs further investigation, although the high primary sequence identity percentage of 81.6% indicates a functional conservation. Furthermore, both the low homology and the low identity percentages of 39.8–43.9% for the included early colonizers *A. naeslundii* and *N. sicca*, implies a unique MdpS occurrence and function for the *Streptococcus* species among the early colonizers of dental surfaces. Although, additional studies are required to determine whether this confers any advantages compared to other early colonizing microorganisms. Moreover, MdpS does not share any domain homology to other characterized O-glycoproteases. Rather MdpS has similarity with tRNA N6-adenosine threonylcarbamoyltransferase complex transferase subunit TsaD as well as a short stretch (amino acid 99–119) with the M22 family with O-sialoglycoprotein endopeptidases. This stresses the importance of characterizing enzymes rather than depending on generic *in silico* annotations.

The physicochemical characterization revealed several dissimilarities comparing MdpS and the previously characterized Mucin Degrading Protease, MdpL (Leo et al., 2023). The inhibition test indicated that MdpS is a serine protease (Figure 2), in contrast to MdpL, which was inhibited by most inhibitor groups (Leo et al., 2023). Further supporting an annotation as a serine protease is the inhibitory effect of Zn^2+^, often required by metalloproteases. MdpL also displayed a strong dependence on a reduced environment but pH - and sodium chloride sensitivity were shown to be modest, hinting at the typical milieu promoting growth of *Limosilactobacillus fermentum*. Thus, when investigating MdpS through a similar perspective, it was expected that MdpS was sensitive to reducing agents, because it correlates well with the high redox potential measured in early oral biofilms, in which *S. oralis* typically exist (Kenney and Ash, 1969; Marquis, 1995). Intriguingly, MdpS was also sensitive to sodium chloride and required a narrow temperature range of around 37°C, which suggests highly limited biological conditions facilitating an efficient hydrolysis. Moreover, the MdpS activity increase influenced by magnesium and especially calcium cations is interesting, because of the chemical composition of unstimulated whole saliva (Rehak et al., 2000). Salivary calcium is crucial for remineralization of the enamel surfaces of teeth (Neel et al., 2016) and it serves as a significant factor in oral biofilm development (Shokeen et al., 2022). Insufficient calcium concentrations would result in a lack of activity, while excessive concentrations may lead to potential interference by other biofilm factors or components. Consequently, calcium levels need to be tightly regulated in order to direct development of an eubiotic oral biofilm.

MdpS is suggested to have a potential immunomodulatory function by targeting two crucial components of the mucosal humoral immune system. MdpS produces substantial hydrolysis of the O-glycosylated IgA1, as well as non-O-glycosylated IgA2 and IgM, and some minor activity toward IgG3 and IgG4 (Supplementary Figure 8; Figure 3B). IgG3 is an O-glycoprotein, whereas IgG4 and IgM are not. Both IgG3 and IgM are known for being less proteolytically resistant, due to their extended- and proteolytically accessible hinge regions. It has been suggested that the included IgG4 in our study, nivolumab, is more protease-sensitive which could explain why MdpS have some activity toward it (Deveuve et al., 2020). Its amino acid composition could also increase the hydrolysis possibilities for MdpS. IgA is the predominant antibody isotype in saliva, with primary roles of maintaining mucosal homeostasis and preventing microorganisms from breaching the mucosal barrier (Corthésy, 2013). Over time, bacteria have evolved enzymatic strategies to evade or deplete this neutralizing function, leading to the identification of IgA proteases in multiple oral streptococcal species, particularly those colonizing dental surfaces (Kilian et al., 1989). Secreted IgA has been shown to inhibit the adherence of oral streptococci to saliva-coated hydroxyapatite; however, this function can be compromised by an IgA1 protease (Reinholdt and Kilian, 1987). MdpS may accomplish a similar result if it is presented extracellularly during early colonization, potentially leading to increased bacterial adhesion. Another scenario is that MdpS, and an IgA protease are acting in concert to promote bacterial survival. A third possibility is that IgA hydrolysis may coincidentally result from the substrate’s sequence characteristics, with a high prevalence of serine and threonine residues, promoting MdpS activity. In conclusion, these findings further emphasize the importance of MdpS and its interactions with the immune system components, which could significantly influence *S. oralis*’ role among early colonizers and the subsequent development of oral biofilms. However, it is essential to note that further studies are necessary, as the results in this paper provide indirect evidence and do not conclusively prove an actual immunomodulatory effect.

MdpS demonstrates a previously unobserved degree of MUC5B degradation, suggesting a novel enzymatic function employed by oral bacteria. In contrast to hydrolysis by MdpL resulting in a low number of MUC5B peptides detected with LC-MS/MS (Leo et al., 2023), MdpS enabled a superior result illustrating substantial degradation (Figure 4). The MUC5B control was partly degraded prior to MdpS incubation, as anticipated, due to its extraction from whole saliva containing enzymatically active bacteria. Nonetheless, this observation does not contradict the fact that the addition of MdpS results in a significant degradation in the N- and C-terminal domains of MUC5B. The MS/MS spectra was extensively investigated, but they lacked diagnostic saccharide oxonium ions from potentially unassigned O-glycopeptides. Also, the use of Ser/Thr cleavage specificities did not produce any new identities, suggesting MdpS is not hydrolyzing similar to previously described O-glycopeptides. Intriguingly, the enzyme exhibits hydrolytic preference within all of the numerous cysteine-rich regions, in which disulfide bridges holds the MUC5B polymers together (Ridley et al., 2014). Consequently, hydrolysis by MdpS could have a considerable structural and functional impact on MUC5B, given that the polymerization is essential for the viscoelastic properties of mucus, allowing it to trap and remove pathogens, debris, and other particles from mucosal surfaces (Thornton et al., 2018). Finally, it is important to note that the increased levels of Ca^2+^ used during the MdpS reaction may induce a more compact conformation in the MUC5B structure (Hughes et al., 2019), potentially leading to a non-native mechanism of action for MdpS.

The amino acid specificity study revealed ambiguous results for MdpS displaying a distinct preference depending on the substrate. In contrast to MdpL, which seemed to have a modest preference for hydrophobic residues in the prim positions (Leo et al., 2023), MdpS favored non-O-glycosylated serine and threonine in close proximity to the hydrolytic site (Figure 5). However, the protease exhibited a distinctive approach depending on the substrate, which may have been influenced by glycosylation. Notably, the amino acid specificity profile of MUC5B was unique, potentially offering a plausible explanation. The presence of lengthy O-glycans in the PST-rich regions might render them proteolytical inaccessible, resulting in hydrolysis occurring primarily in the cysteine-rich regions (Figure 4). Concludingly, the amino acid specificity aligns well with the recently characterized O-glycoproteases (Shon et al., 2021), with Ser/Thr on either side of the hydrolytic site. However, a major difference is that MdpS rarely hydrolyze immediately N- or C-terminally of an O-glycosylated site (Figure 4; Supplementary Figures 7, 8), but still prefers O-glycosylated substrates (Figure 3). This preference has also been shown for the ToxR-activated gene A (TagA) from *Vibrio cholerae* exhibiting a selective activity for mucin substrates (Szabady et al., 2011), without requiring mucin-type O-glycosylation for hydrolysis (Shon et al., 2020). In contrast to TagA, which belongs in the MEROPS clan MA(M), M66 family, MdpS does not possess the distinctive sequence of HEXGHXXXGXGH, and its activity is not dependent on zinc but rather on other cations. Furthermore, the sequence homology was only 22.5%, suggesting the TagA activity toward salivary mucins together with the mechanism of action are the only similarities between the two enzymes. The characterization of MdpS suggests the identification of a previously undescribed enzyme family that has some resemblance to the M66 family. This finding further underscores the existing gaps in our understanding of MUC5B degradation and highlights the need for the identification of other novel enzymes.

The autoproteolytic enzyme fragment observed after 4 h (Supplementary Table 1) could represent an activated form of MdpS, possibly serving as the catalyst for subsequent self-activation, continued autoproteolysis, and/or substrate hydrolysis. Alternatively, this processing and activation could occur through other bacterial or host proteases in a native physicochemical biofilm setting, hydrolyzing specific N- and/or C-terminal signaling peptides of MdpS (Kasperkiewicz, 2021). Considering that the tested MdpS was recombinantly and not natively produced, it suggests that MdpS may be less effective in the initial stages of the reaction. The autoproteolysis data indicates the presence of an extended pre-activation phase before achieving efficient substrate hydrolysis. It should however be noted that autoproteolysis might originate from improper folding during recombinant expression, potentially leading to an artifact that may not accurately reflect the native conditions. These findings are consistent with a previously proposed theory that suggests stringent regulation of serine protease activity across three pivotal levels, where deliberate activation of inactive zymogens through specific posttranslational modifications, is the second regulatory level (Hedstrom, 2002; Verdoes and Verhelst, 2016). The first level, as detailed in the preceding paragraph, pertains to protease expression. The third level encompasses the inactivation of serine proteases by endogenous serine inhibitors found in saliva, such as the secretory leukocyte protease inhibitor and α1-antitrypsin (Pederson et al., 1995; Jacobsen et al., 2008). Apart from these inhibitors, bacteria residing within the biofilm can also release inhibitory substances, significantly affecting their fellow bacteria, either as collaborators or competitors. Precise control of MUC5B degradation is imperative to prevent untimely and unsanctioned release of carbon and nitrogen sources and to preserve the protective function of MUC5B. Without its polymeric form, it could potentially be advantageous to pathogenic microorganisms. Furthermore, regulated nutrient release not only promotes the growth of *S. oralis* but also plays a pivotal role in facilitating cross-feeding to other biofilm microorganisms and regulating biofilm succession, thus ensuring the maintenance of a eubiotic oral environment.

In conclusion, understanding the interplay between *S. oralis* and MUC5B has implications for oral health and disease, because a dysregulation of this interaction could potentially contribute to dysbiosis. Furthermore, unraveling the molecular mechanisms underlying this interaction could pave the way for targeted interventions aimed at modulating oral biofilm composition and mitigating oral diseases. The discovery and characterization of MdpS have accentuated the necessity in continuing studies on the molecular mechanisms involved in mucin degradation, because of their potential impact as modulators of the oral microbiome which could impact the overall health of the oral cavity. Furthermore, these MdpS data have challenged the currently accepted model for MUC5B-degradation, by not requiring O-glycan removal prior to extensive peptide backbone hydrolysis.

## Data availability statement

The datasets presented in this study can be found in online repositories. The names of the repository/repositories and accession number(s) can be found at: https://www.ebi.ac.uk/pride/archive/, PXD046954 and PXD046810.

## Author contributions

FL: Writing – original draft, Writing – review & editing. RL: Writing – review & editing. KT: Writing – review & editing. JN: Writing – review & editing. GS: Writing – review & editing. CW: Writing – review & editing.

## References

[ref1] Chahal G. Quintana-Hayashi M. P. Gaytán M. O. Benktander J. Padra M. King S. J.. (2022). *Streptococcus oralis* employs multiple mechanisms of salivary mucin binding that differ between strains. Front. Cell. Infect. Microbiol. 12, 1–14. doi: 10.3389/fcimb.2022.889711, PMID: 35782137 PMC9247193

[ref2] Corthésy B. (2013). Multi-faceted functions of secretory IgA at mucosal surfaces. Front. Immunol. 4, 1–11. doi: 10.3389/fimmu.2013.00185, PMID: 23874333 PMC3709412

[ref3] Crouch L. I. Liberato M. V. Urbanowicz P. A. Baslé A. Lamb C. A. Stewart C. J.. (2020). Prominent members of the human gut microbiota express endo-acting O-glycanases to initiate mucin breakdown. Nat. Commun. 11:4017. doi: 10.1038/s41467-020-17847-5, PMID: 32782292 PMC7419316

[ref4] Derrien M. Vaughan E. E. Plugge C. M. De Vos W. M. (2004). *Akkermansia muciniphila* gen. Nov., sp. nov., a human intestinal mucin-degrading bacterium. Int. J. Syst. Evol. Microbiol. 54, 1469–1476. doi: 10.1099/ijs.0.02873-0 15388697

[ref5] Deveuve Q. Lajoie L. Barrault B. Thibault G. (2020). The proteolytic cleavage of therapeutic monoclonal antibody hinge region: more than a matter of subclass. Front. Immunol. 11, 1–12. doi: 10.3389/fimmu.2020.00168, PMID: 32117299 PMC7026020

[ref6] Diaz P. I. Chalmers N. I. Rickard A. H. Kong C. Milburn C. L. Palmer R. J.. (2006). Molecular characterization of subject-specific oral microflora during initial colonization of enamel. Appl. Environ. Microbiol. 72, 2837–2848. doi: 10.1128/AEM.72.4.2837-2848.2006, PMID: 16597990 PMC1449052

[ref7] Frenkel E. S. Ribbeck K. (2015). Salivary mucins protect surfaces from colonization by cariogenic bacteria. Appl. Environ. Microbiol. 81, 332–338. doi: 10.1128/AEM.02573-14, PMID: 25344244 PMC4272720

[ref8] Hedstrom L. (2002). Serine protease mechanism and specificity. Chem. Rev. 102, 4501–4524. doi: 10.1021/cr000033x 12475199

[ref9] Hughes G. W. Ridley C. Collins R. Roseman A. Ford R. Thornton D. J. (2019). The MUC5B mucin polymer is dominated by repeating structural motifs and its topology is regulated by calcium and pH. Sci. Rep. 9, 17350–17313. doi: 10.1038/s41598-019-53768-0, PMID: 31758042 PMC6874590

[ref10] Jacobsen L. C. Sørensen O. E. Cowland J. B. Borregaard N. Theilgaard-Mönch K. (2008). The secretory leukocyte protease inhibitor (SLPI) and the secondary granule protein lactoferrin are synthesized in myelocytes, colocalize in subcellular fractions of neutrophils, and are coreleased by activated neutrophils. J. Leukoc. Biol. 83, 1155–1164. doi: 10.1189/jlb.0706442, PMID: 18285402

[ref11] JinC.KennyD. T.SkoogE. C.PadraM.AdamczykB.VitizevaV.. (2017). Structural diversity of human gastric mucin glycans. Mol. Cell. Proteomics 16, 743–758. doi: 10.1074/mcp.M117.067983, PMID: 28461410

[ref12] Kasperkiewicz P. (2021). Peptidyl activity-based probes for imaging serine proteases. Front. Chem. 9, 1–21. doi: 10.3389/fchem.2021.639410, PMID: 33996745 PMC8117214

[ref13] Kenney E. B. Ash M. M. (1969). Oxidation reduction potential of developing plaque, periodontal pockets and gingival sulci. J. Periodontol. 40, 630–633. doi: 10.1902/jop.1969.40.11.630, PMID: 5260618

[ref14] Kilian M. Reinholdt J. Nyvad B. Frandsen E. V. G. Mikkelsen L. (1989). IgA1 proteases of oral streptococci: ecological aspects. Immunol. Investig. 18, 161–170. doi: 10.3109/08820138909112235 2659509

[ref15] Kim Y. J. Borsig L. Han H. L. Varki N. M. Varki A. (1999). Distinct selectin ligands on colon carcinoma mucins can mediate pathological interactions among platelets, leukocytes, and endothelium. Am. J. Pathol. 155, 461–472. doi: 10.1016/S0002-9440(10)65142-5, PMID: 10433939 PMC1866847

[ref16] Lamont R. J. Koo H. Hajishengallis G. (2018). The oral microbiota: dynamic communities and host interactions. Nat. Rev. Microbiol. 16, 745–759. doi: 10.1038/s41579-018-0089-x, PMID: 30301974 PMC6278837

[ref17] Leo F. Svensäter G. Lood R. Wickström C. (2023). Characterization of a highly conserved MUC5B-degrading protease, MdpL, from Limosilactobacillus fermentum. Front. Microbiol. 14, 1–13. doi: 10.3389/fmicb.2023.1127466, PMID: 36925480 PMC10011156

[ref18] Madsen J. B. Svensson B. Abou Hachem M. Lee S. (2015). Proteolytic degradation of bovine submaxillary mucin (BSM) and its impact on adsorption and lubrication at a hydrophobic surface. Langmuir 31, 8303–8309. doi: 10.1021/acs.langmuir.5b01281, PMID: 26153254

[ref19] Marquis R. E. (1995). Oxygen metabolism, oxidative stress and acid-base physiology of dental plaque biofilms. J. Ind. Microbiol. 15, 198–207. doi: 10.1007/BF01569826, PMID: 8519478

[ref20] Marsh P. D. (1994). Microbial ecology of dental plaque and its significance in health and disease. Adv. Dent. Res. 8, 263–271. doi: 10.1177/08959374940080022001, PMID: 7865085

[ref21] Marsh P. D. (2010). Microbiology of dental plaque biofilms and their role in oral health and caries. Dent. Clin. N. Am. 54, 441–454. doi: 10.1016/j.cden.2010.03.002 20630188

[ref22] Neel E. A. A. Aljabo A. Strange A. Ibrahim S. Coathup M. Young A. M.. (2016). Demineralization–remineralization dynamics in teeth and bone. Int. J. Nanomedicine 11, 4743–4763. doi: 10.2147/IJN.S107624, PMID: 27695330 PMC5034904

[ref23] Nobbs A. H. Lamont R. J. Jenkinson H. F. (2009). Streptococcus adherence and colonization. Microbiol. Mol. Biol. Rev. 73, 407–450. doi: 10.1128/mmbr.00014-09, PMID: 19721085 PMC2738137

[ref24] Nyvad B. Kilian M. (1990). Comparison of the initial streptococcal microflora on dental enamel in caries-active and in caries-inactive individuals. Caries Res. 24, 267–272. doi: 10.1159/000261281, PMID: 2276164

[ref25] Okuda K. Chen G. Subramani D. B. Wolf M. Gilmore R. C. Kato T.. (2019). Localization of secretory mucins MUC5AC and MUC5B in normal/healthy human airways. Am. J. Respir. Crit. Care Med. 199, 715–727. doi: 10.1164/rccm.201804-0734OC, PMID: 30352166 PMC6423099

[ref26] Pederson E. D. Stanke S. R. Whitener S. J. Sebastiani P. T. Lamberts B. L. Turner D. W. (1995). Salivary levels of α2-macroglobulin, α1-antitrypsin, C-reactive protein, cathepsin G and elastase in humans with or without destructive periodontal disease. Arch. Oral Biol. 40, 1151–1155. doi: 10.1016/0003-9969(95)00089-5 8850655

[ref27] Perez-Riverol Y. Bai J. Bandla C. García-Seisdedos D. Hewapathirana S. Kamatchinathan S.. (2022). The PRIDE database resources in 2022: a hub for mass spectrometry-based proteomics evidences. Nucleic Acids Res. 50, D543–D552. doi: 10.1093/nar/gkab1038, PMID: 34723319 PMC8728295

[ref28] Rehak N. N. Cecco S. A. Csako G. (2000). Biochemical composition and electrolyte balance of “unstimulated” whole human saliva. Clin. Chem. Lab. Med. 38, 335–343. doi: 10.1515/CCLM.2000.049, PMID: 10928655

[ref29] Reinholdt J. Kilian M. (1987). Interference of IgA protease with the effect of secretory IgA on adherence of Oral streptococci to saliva-coated hydroxyapatite. J. Dent. Res. 66, 492–497. doi: 10.1177/00220345870660021801, PMID: 3040826

[ref30] Richards V. P. Palmer S. R. Bitar P. D. P. Qin X. Weinstock G. M. Highlander S. K.. (2014). Phylogenomics and the dynamic genome evolution f the genus streptococcus. Genome Biol. Evol. 6, 741–753. doi: 10.1093/gbe/evu048, PMID: 24625962 PMC4007547

[ref31] Ridley C. Kouvatsos N. Raynal B. D. Howard M. Collins R. F. Desseyn J. L.. (2014). Assembly of the respiratory mucin MUC5B a new model for a gel-forming mucin. J. Biol. Chem. 289, 16409–16420. doi: 10.1074/jbc.M114.566679, PMID: 24778189 PMC4047408

[ref32] Shokeen B. Pham E. Esfandi J. Kondo T. Okawa H. Nishimura I.. (2022). Effect of calcium ion supplementation on Oral microbial composition and biofilm formation in vitro. Microorganisms 10. doi: 10.3390/microorganisms10091780, PMID: 36144381 PMC9500923

[ref33] Shon D. J. Kuo A. Ferracane M. J. Malaker S. A. (2021). Classification, structural biology, and applications of mucin domain-targeting proteases. Biochem. J. 478, 1585–1603. doi: 10.1042/BCJ20200607, PMID: 33909028

[ref34] Shon D. J. Malaker S. A. Pedram K. Yang E. Krishnan V. Dorigo O.. (2020). An enzymatic toolkit for selective proteolysis, detection, and visualization of mucin-domain glycoproteins. Proc. Natl. Acad. Sci. U. S. A. 117, 21299–21307. doi: 10.1073/pnas.2012196117, PMID: 32817557 PMC7474620

[ref35] Siciliano R. A. Lippolis R. Mazzeo M. F. (2019). Proteomics for the investigation of surface-exposed proteins in probiotics. Front. Nutr. 6:52. doi: 10.3389/fnut.2019.00052, PMID: 31069232 PMC6491629

[ref36] Szabady R. L. Yanta J. H. Halladin D. K. Schofield M. J. Welch R. A. (2011). TagA is a secreted protease of *Vibrio cholerae* that specifically cleaves mucin glycoproteins. Microbiology 157, 516–525. doi: 10.1099/MIC.0.044529-0, PMID: 20966091 PMC3090133

[ref37] Taleb V. Liao Q. Narimatsu Y. García-García A. Compañón I. Borges R. J.. (2022). Structural and mechanistic insights into the cleavage of clustered O-glycan patches-containing glycoproteins by mucinases of the human gut. Nat. Commun. 13, 4324–4315. doi: 10.1038/s41467-022-32021-9, PMID: 35882872 PMC9325726

[ref38] Teles F. R. Teles R. P. Uzel N. G. Song X. Q. Torresyap G. Socransky S. S.. (2012). Early microbial succession in redeveloping dental biofilms in periodontal health and disease. J. Periodontal Res. 47, 95–104. doi: 10.1111/j.1600-0765.2011.01409.x, PMID: 21895662 PMC3253172

[ref39] Thomsson K. A. Prakobphol A. Leffler H. Reddy M. S. Levine M. J. Fisher S. J.. (2002). The salivary mucin MG1 (MUC5B) carries a repertoire of unique oligosaccharides that is large and diverse. Glycobiology 12, 1–14. doi: 10.1093/glycob/12.1.1, PMID: 11825880

[ref40] Thornton D. J. Sharpe C. Ridley C. (2018). Intracellular processing of human secreted polymeric airway mucin. Ann. Am. Thorac. Soc. 15, S154–S158. doi: 10.1513/AnnalsATS.201802-143AW, PMID: 30431345 PMC6276985

[ref41] van Der Hoeven J. S. van Den Kieboom C. W. A. Camp P. J. M. (1990). Utilization of mucin by oral Streptococcus species. Antonie Van Leeuwenhoek 57, 165–172. doi: 10.1007/BF00403951, PMID: 2321937

[ref42] Verdoes M. Verhelst S. H. L. (2016). Detection of protease activity in cells and animals. Biochim. Biophys. Acta, Proteins Proteomics 1864, 130–142. doi: 10.1016/j.bbapap.2015.04.029 25960278

[ref43] Wickström C. Davies J. R. Eriksen G. V. Veerman E. C. I. Carlstedt I. (1998). MUC5B is a major gel-forming, oligomeric mucin from human salivary gland, respiratory tract and endocervix: identification of glycoforms and C-terminal cleavage. Biochem. J. 334, 685–693. doi: 10.1042/bj3340685, PMID: 9729478 PMC1219739

[ref44] Wickström C. Svensäter G. (2008). Salivary gel-forming mucin MUC5B - a nutrient for dental plaque bacteria. Oral Microbiol. Immunol. 23, 177–182. doi: 10.1111/j.1399-302X.2007.00407.x, PMID: 18402602

[ref45] Winchester B. (2005). Lysosomal metabolism of glycoproteins. Glycobiology 15, 1R–15R. doi: 10.1093/glycob/cwi041 15647514

[ref46] Zaretsky J. Z. Wreschner D. H. (2013). “Gel-forming mucin MUC5B” in Mucins – Potential regulators of cell functions volume title: Gel-forming and soluble mucins (Bentham science). Bentham Science Publisher. 246–315.

