# Supplementary material for: Characterization of MdpS: an in-depth analysis of a MUC5B-degrading protease from Streptococcus oralis
Source: Front Microbiol. 2024 Jan 18;15:1340109. doi: 10.3389/fmicb.2024.1340109 (PMC10830703; doi:10.3389/fmicb.2024.1340109)
Supplement: Supplementary file 2 [file Data_Sheet_1.PDF]

Supplementary Material

Characterization of MdpS: An In-depth Analysis of a MUC5B-Degrading Protease from *Streptococcus oralis*

Fredrik Leo*, Rolf Lood, Kristina A. Thomsson, Jonas Nilsson, Gunnel Svensäter, Claes Wickström

*** Correspondence:** Fredrik Leo: fredrik.leo@mau.se

```
>MdpS
MKDRYILAFETSCDETSVAVLKNDDELLSNVIASQIESHKRFGGVVPEVASRHHVEVITACIEEALAE
AGITEEDVTAVAVTYGPGLVGALLVGLSAAKAFAWAHGLPLIPVNHMAGHLMAAQSVEPLEFPLLALL
VSGGHTELVYVSEAGDYKIVGETRDDAVGEAYDKVGRVMGLTYPAGREIDELAHKGQDVYDFPRAMIK
EDNLEFSFSGLKSAFINLHHNAEQKGESLSKEDLSASFQAAVMDILMAKTKKALGEYPVKTLVVAGGV
AANKGLRERLAAEITDVKVIIPPLRLCGDNAGMIAYASVSEWNKGNFAGLDLNAKPSLAFDTMEGSGH
HHHHH
```

Supplementary figure 1: FASTA sequence of MdpS (GenBank accession number WP_084852800.1) with C-terminal GSG and 6xHis-tag.

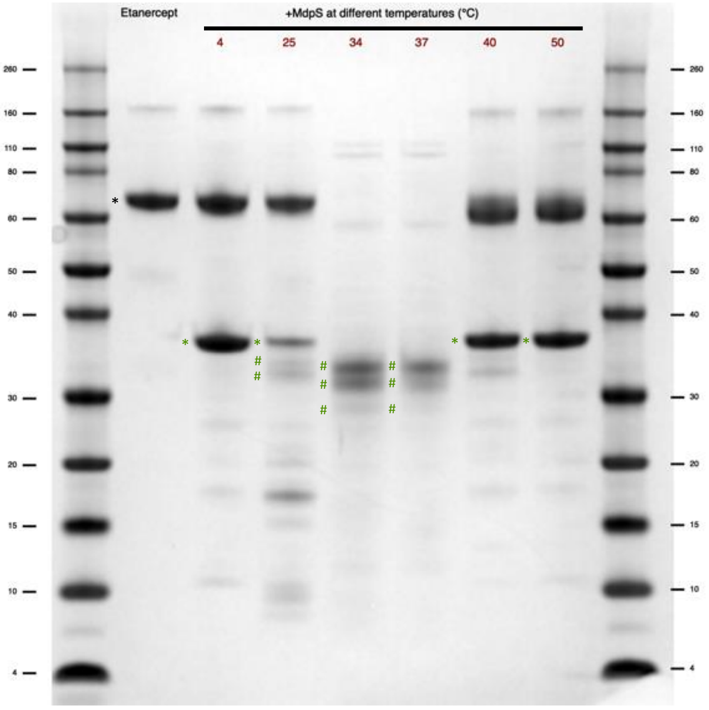

Supplementary figure 2: A representative gel assessing the effect of temperature on MdpS activity. Intact substrate (black asterisk: *), intact MdpS (green asterisk: *), and MdpS-generated fragments (green hashtag: #).

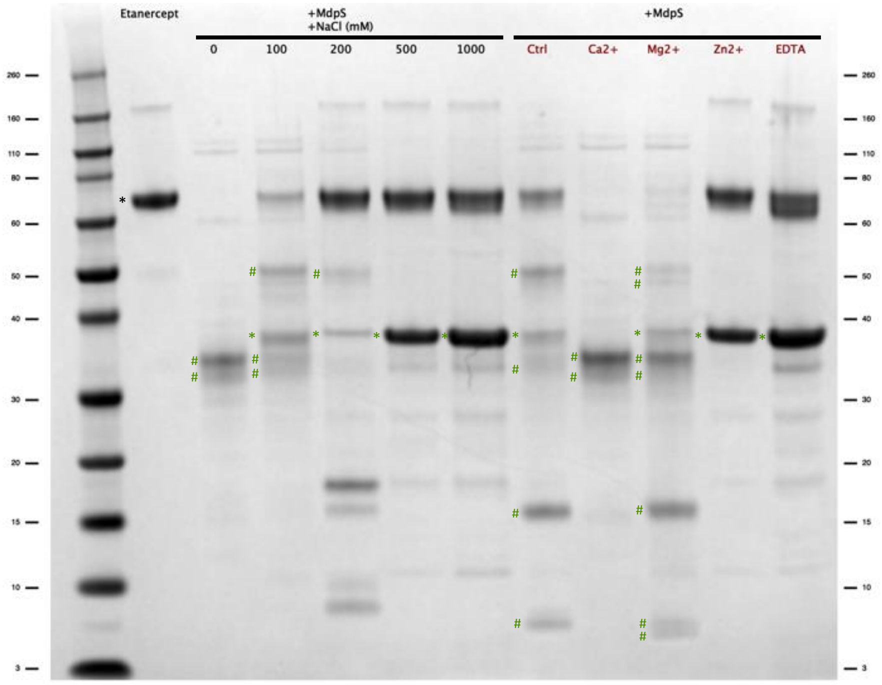

Supplementary figure 3: A representative gel assessing the effect of ions, EDTA and NaCl on MdpS activity. Intact substrate (black asterisk: *), intact MdpS (green asterisk: *), and MdpS-generated fragments (green hashtag: #).

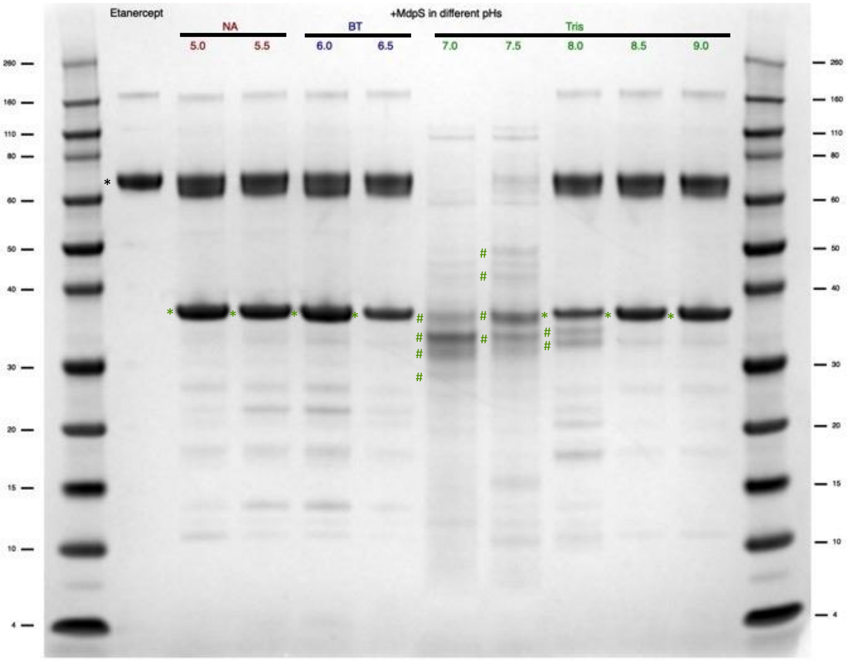

Supplementary figure 4: A representative gel assessing the effect of pH on MdpS activity. Intact substrate (black asterisk: *), intact MdpS (green asterisk: *), and MdpS-generated fragments (green hashtag: #).

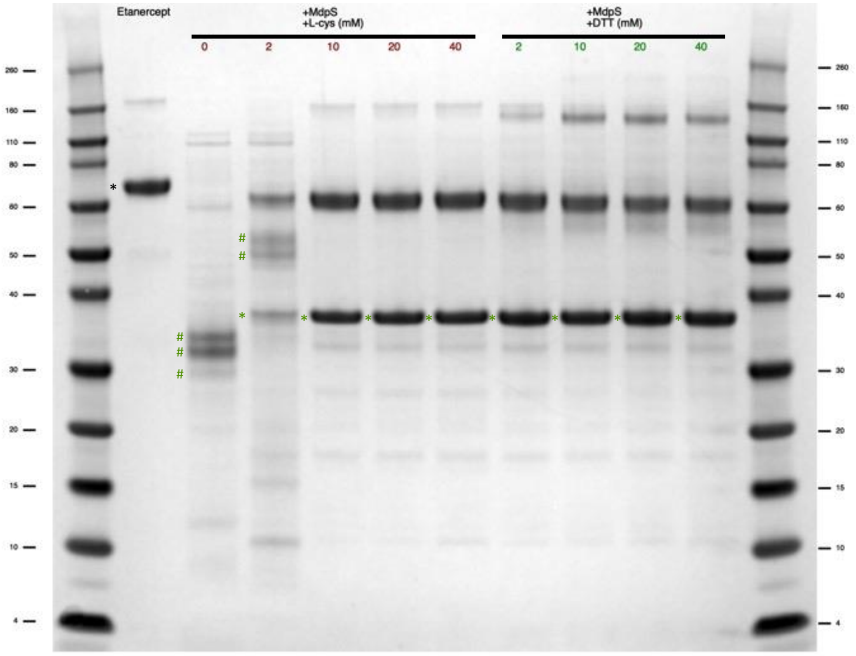

Supplementary figure 5: A representative gel assessing the effect of reducing agents L-cys and DTT on MdpS activity. Intact substrate (black asterisk: *), intact MdpS (green asterisk: *), and MdpS-generated fragments (green hashtag: #).

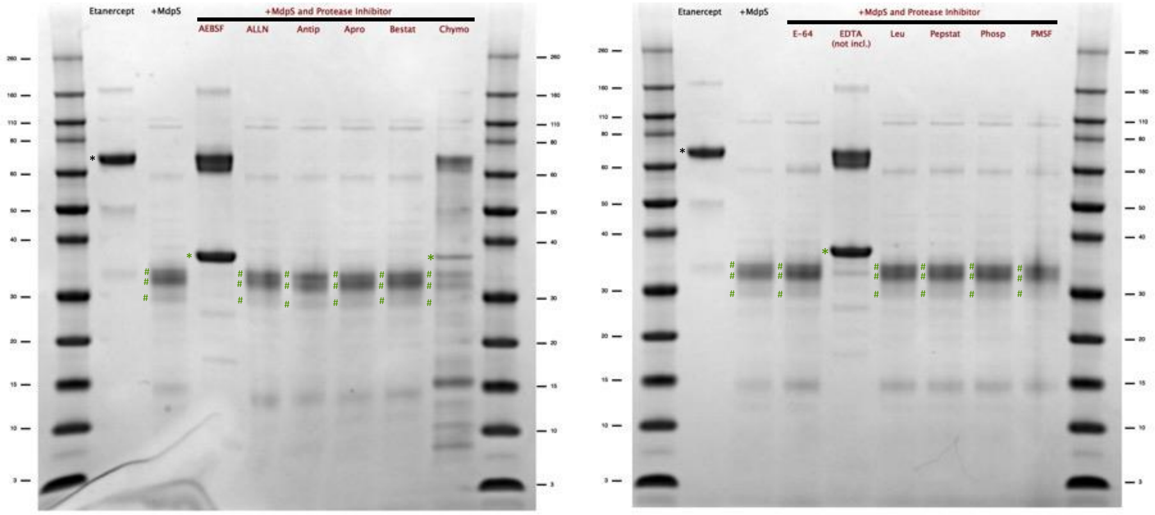

Supplementary figure 6: A representative gel assessing the effect of protease inhibitors on MdpS activity. Intact substrate (black asterisk: *), intact MdpS (green asterisk: *), and MdpS-generated fragments (green hashtag: #).

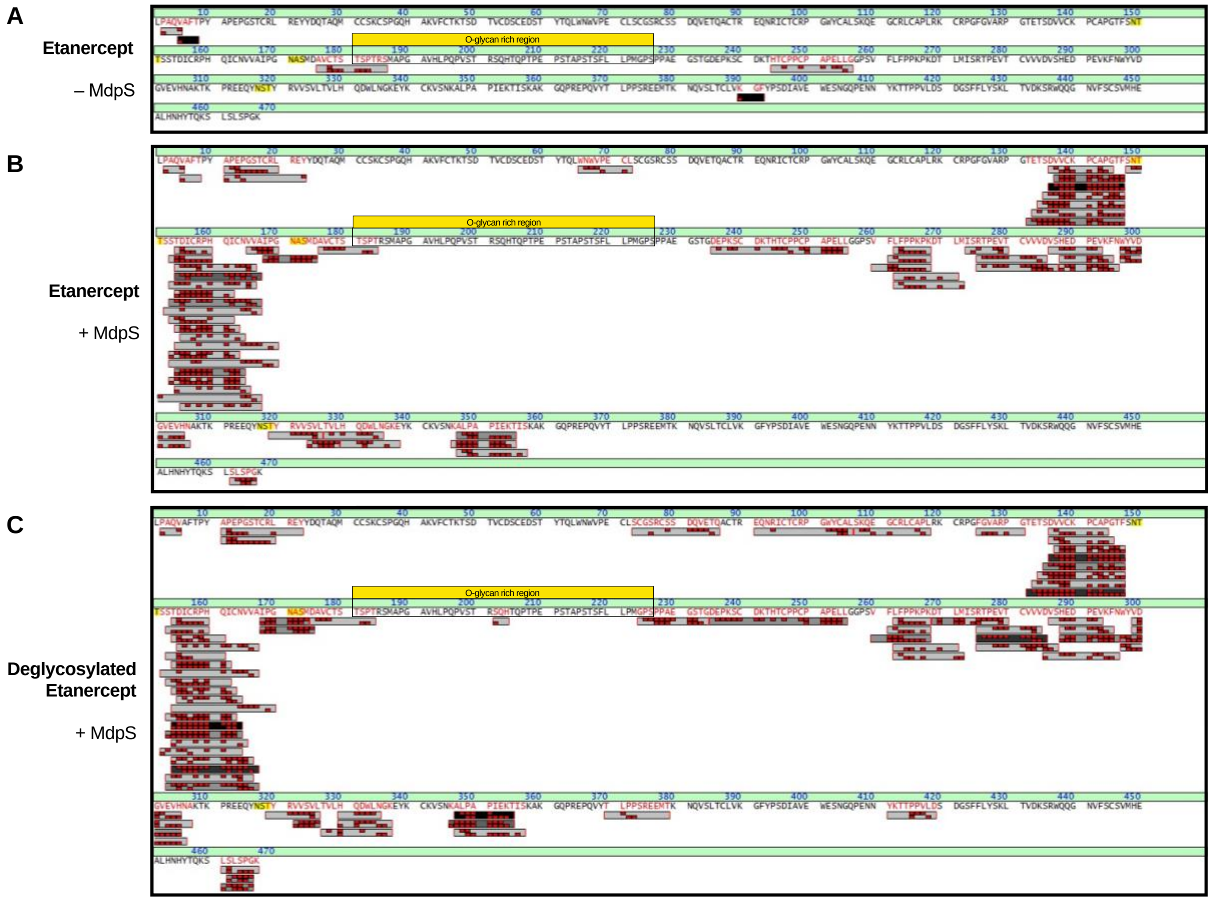

Supplementary figure 7: Overview of Etanercept hydrolysis by MdpS analyzed by LC-MS^2. Peptide map of Etanercept (**A**) without MdpS, (**B**) with MdpS, and (**C**) deglycosylated prior to incubation with MdpS. The data shows that MdpS extensively hydrolyzes Etanercept both N- and C-terminal of the O-glycan rich region (amino acid position 181-226), whereas a low number of peptides can be found within the O-glycan rich hinge region. The peptide maps are highly similar comparing the hydrolysis of intact- and pre-deglycosylated Etanercept, implying that MdpS activity are not necessitated by the presence of O-glycans. All detected peptides are depicted with greyscale boxes, where the darker colors have higher scores according to the analytical software. Red squares inside the grey boxes are sites where b – and/or y ions have a match, which typically increases the peptide score.

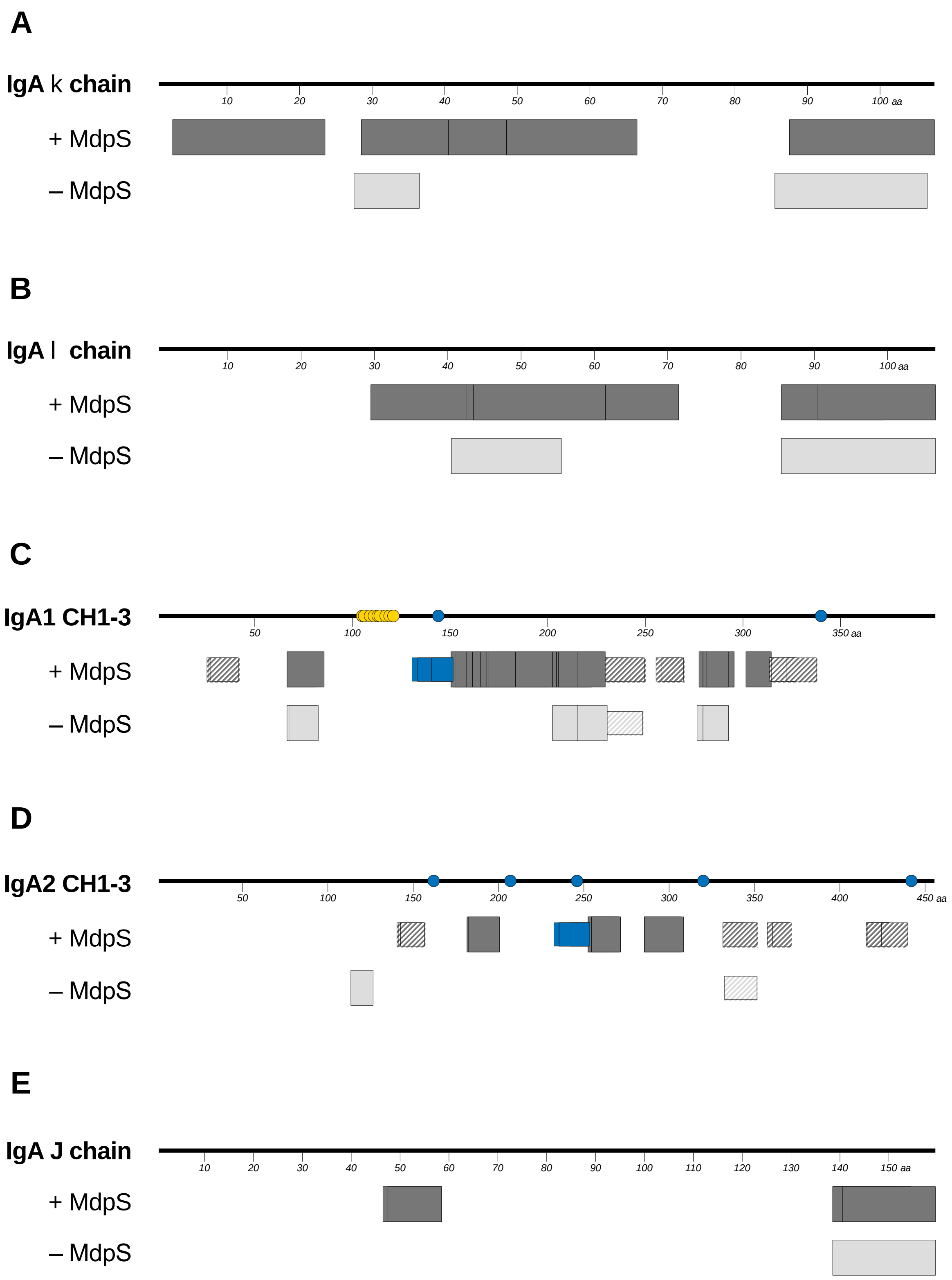

Supplementary figure 8: Schematic peptide map of the degradation of salivary IgA analyzed by NanoLC-MS. The graph shows the identified peptides of the IgA **(A)** kappa, **(B)** lambda chain, the constant heavy chains of **(C)** IgA1, **(D)** and IgA2, and **(E)** the J chain after incubation ± MdpS. The yellow and blue circles represent putative O- or N-glycan sites on the IgA fragments respectively, whereas the blue boxes show the identified N-glycan peptides in the MdpS sample. Filled grey boxes displays the detected peptides and the striped boxes are peptides that analytically cannot be discriminated whether they belong to the IgA1 or the IgA2 constant heavy chains.

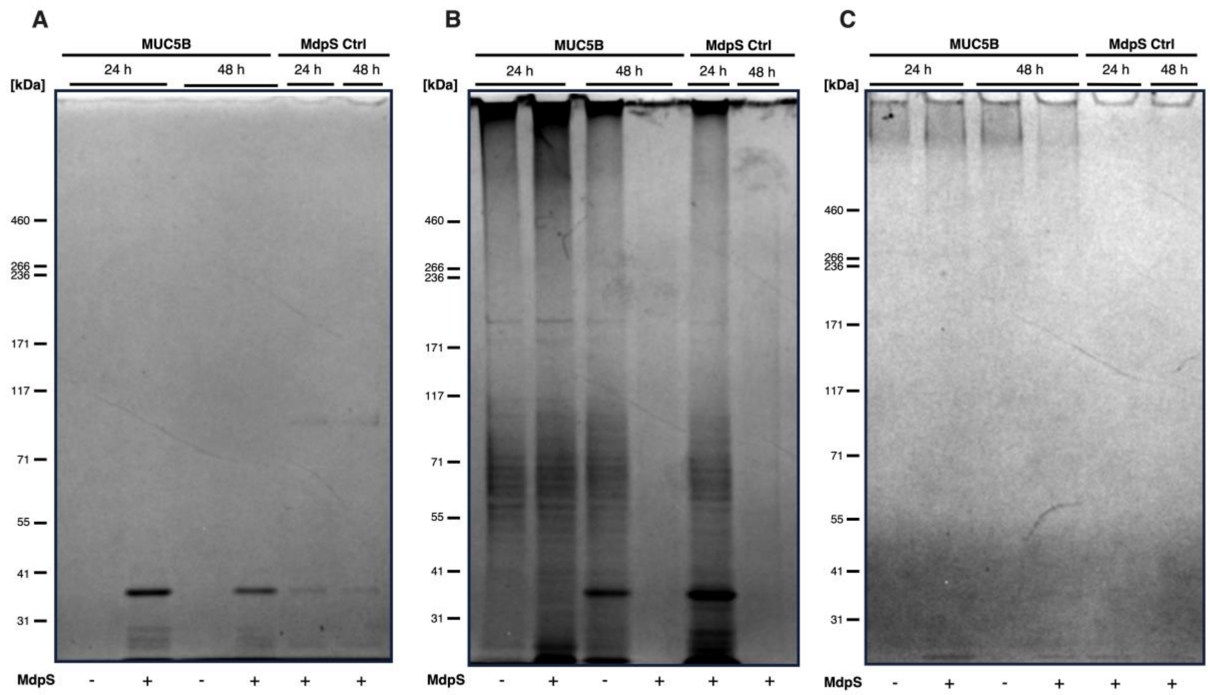

Supplementary figure 9: MdpS-mediated MUC5B degradation. After 24-hour, and 48-hour incubation with MdpS, reduced MUC5B was analyzed using SDS-PAGE followed by (A) Coomassie, (**B**) Silver, and (**C**) PAS staining, revealing a decrease in band intensity corresponding to fragmented MUC5B.

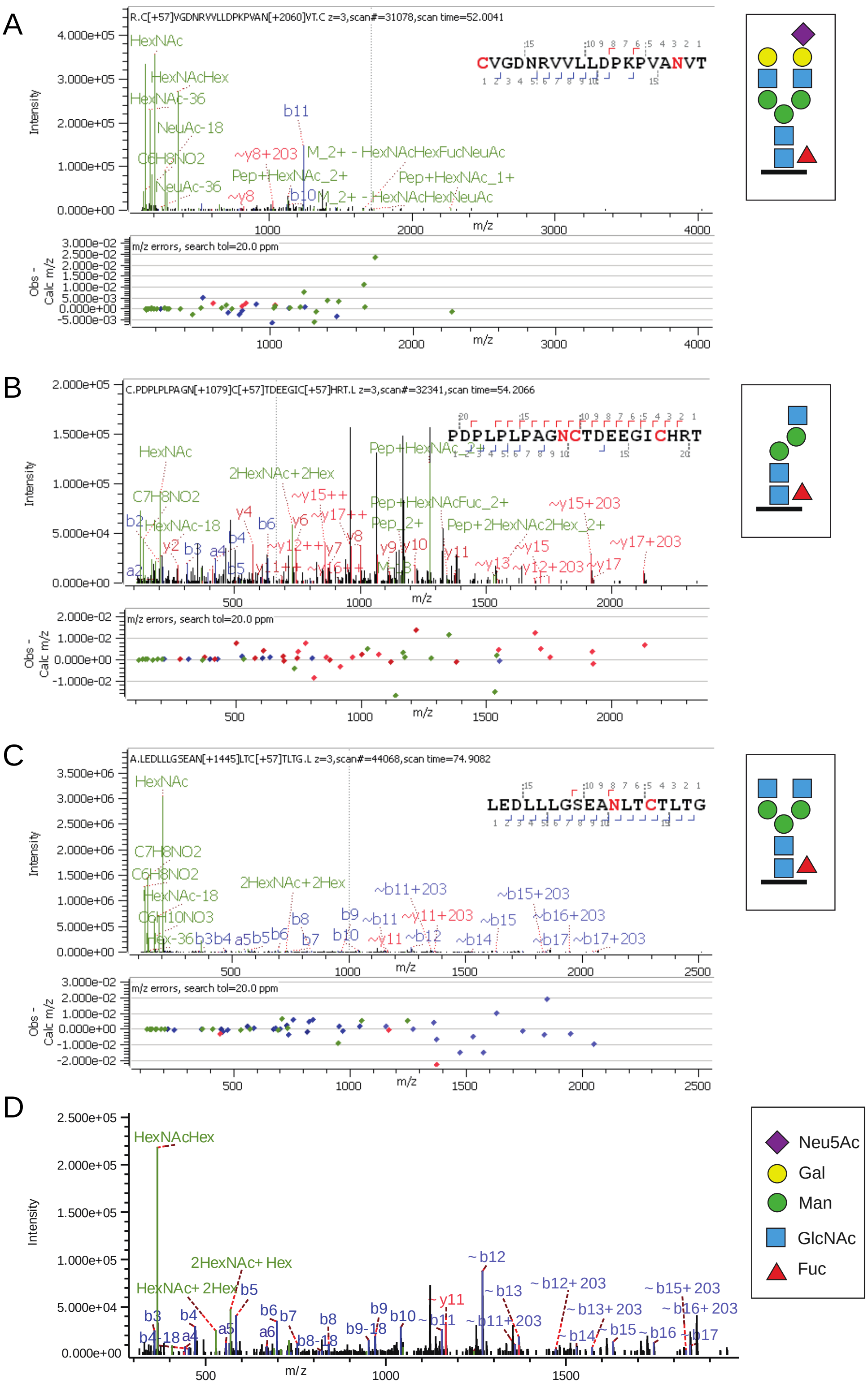

Supplementary figure 10: Glycoproteomic analysis using the Byonic software. (**A**) The Byonic-annotated MS2 spectrum of C.VGDNRVVLLDPKPVANVT.C carrying a HexNAc(4)Hex(5)Fuc(1)NeuAc(1) N-glycan (boxed structure) from residues 5031-5048 of the MUC5B sequence. (**B**) The Byonic-annotated MS2 spectrum of C.PDPLPLPAGNCTDEEGICHRT.L carrying a HexNAc(3)Hex(2)Fuc(1) N-glycan (boxed structure) from residues 245-265 of the MUC5B sequence. (**C**) The Byonic-annotated MS2 spectrum of A.LEDLLLGSEANLTCTLTG.L carrying a HexNAc(4)Hex(3)Fuc(1) N-glycan

(boxed structure) from residues 134-151 of the IgA1 sequence. **(D)** The *m/z* 300-2000 expansion of the spectra in panel C to verify that the annotated ions do not originate from noise peaks. A majority of the most intense ions are matched to the sequence. The Asn N-glycosylation sites are underlined. Cysteines are carbamido-derivatized (marked red). Identified peptide fragmentation b- and y-ions (marked blue and red, respectively) are indicated in relation to the peptide sequences. Identified saccharide oxonium ions and glycosidic fragmentations are indicated (marked green). The mass accuracy of all identified ions are indicated below the spectra (MS2 search tolerance = 20 ppm). The Byonic viewer files, tables of spectral matches and MS raw files are available via the PRIDE repository for this project (dataset identifier PXD046810).

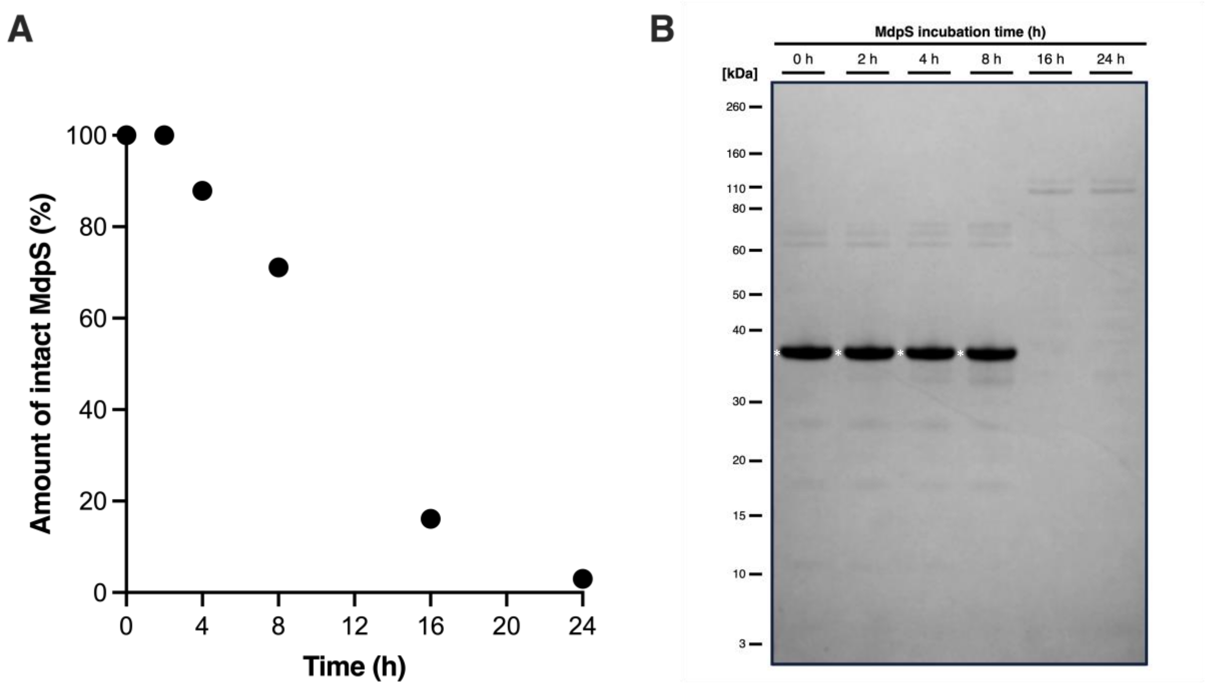

Supplementary figure 11: Amount of intact MdpS after 0-24 h autoproteolysis analyzed with **(A)** LC-MS, and **(B)** SDS-PAGE. Detailed information on the identified fragments is summarized in Suppl. Table 1 below. Intact MdpS is marked with white asterisks (*).

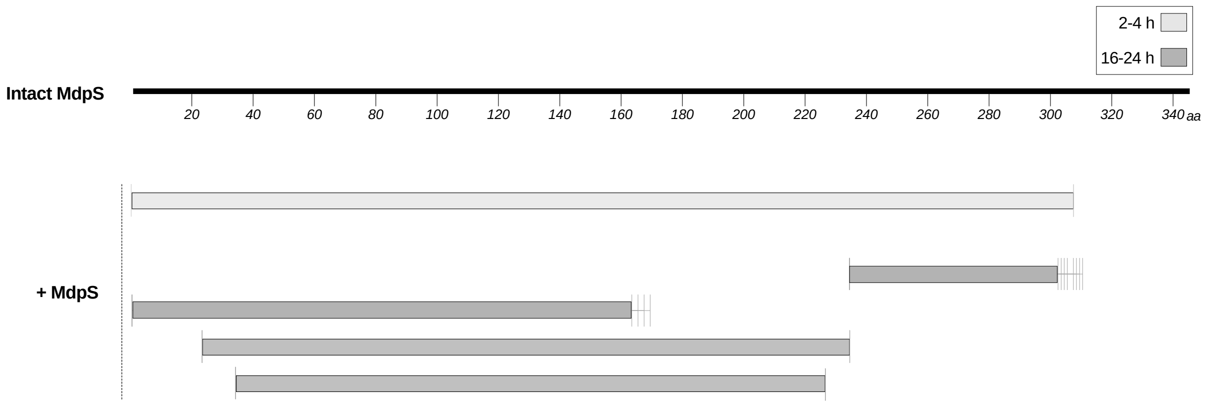

Supplementary figure 12: Protein fragments detected by LC-MS after autoproteolytic hydrolysis of MdpS. The upper panel displays intact MdpS (345 aa), whereas the bottom panel illustrates the protein fragments generated by autoproteolysis after 2-4 and 16-24 h. Detailed information on the identified fragments is summarized in Suppl. Table 1 below.

Supplementary Table 1: Summary of all detected intact and fragmented MdpS after autoproteolysis

Protein fragment #	MW calc.	MW exp.	Deviation (Da)	Time Point (h)	Intensity	MdpS fragment
**1**	36955.0222	36954.8	0.2	0	260165	Intact MdpS
				2	263236	
				4	228729	
				8	185019	
				16	41969	
				24	7704	
**2**	32776.1548	32775.9	0.3	4	3423	1-307
	32776.1675			8	5661	
**3**	7205.0090	7205.0	0.0	16	112071	235-302
	7204.9976			24	3722	
**4**	7276.0325	7276.0	0.0	16	262645	235-303
	7276.0265			24	9097	
**5**	7648.2250	7648.2	0.0	16	159832	235-307
	7648.2139			24	4235	
**6**	7333.0475	7333.0	0.0	16	69735	235-304
	7333.0307			24	1406	
**7**	7464.1096	7464.1	0.0	16	61260	235-305
**8**	7811.2959	7811.2	0.1	16	127377	235-308
	7811.2723			24	2700	
**9**	7882.3230	7882.3	0.0	16	25905	235-309
**10**	7969.3452	7969.3	0.0	16	17120	235-310
**11**	20515.6540	20515.4	0.3	16	3805	35-226
**12**	17238.9093	17238.8	0.1	16	14914	1-163
	17238.8798			24	1880	
**13**	17394.9946	17394.9	0.1	16	23189	1-165
	17394.9850			24	2582	
**14**	17595.0724	17595.0	0.1	16	1900	1-167
**15**	17873.1719	17873.1	0.1	16	3993	1-169
	17873.1616			24	1871	
**16**	22530.4048	22530.4	0.0	24	2076	24-234

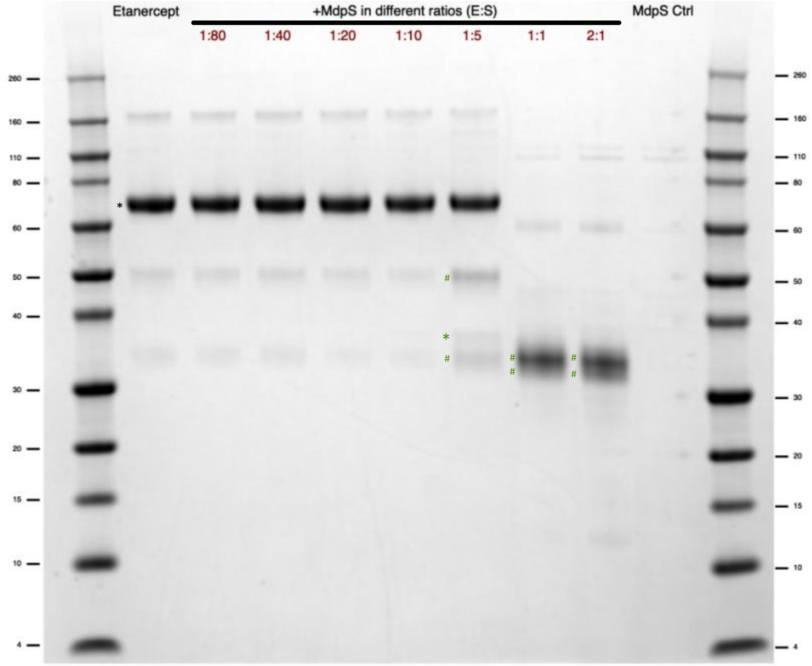

Supplementary figure 13: Enzyme-Substrate ratio test analyzed by SDS-PAGE. MdpS reaction with Etanercept, with enzyme-substrate ratios ranging from 1:80 to 2:1 (w/v), shown with a distinct protein migration pattern. Substrate and enzyme controls are shown on either side of the hydrolytic samples. Intact substrate (black asterisk: *), intact MdpS (green asterisk: *), and MdpS-generated fragments (green hashtag: #).

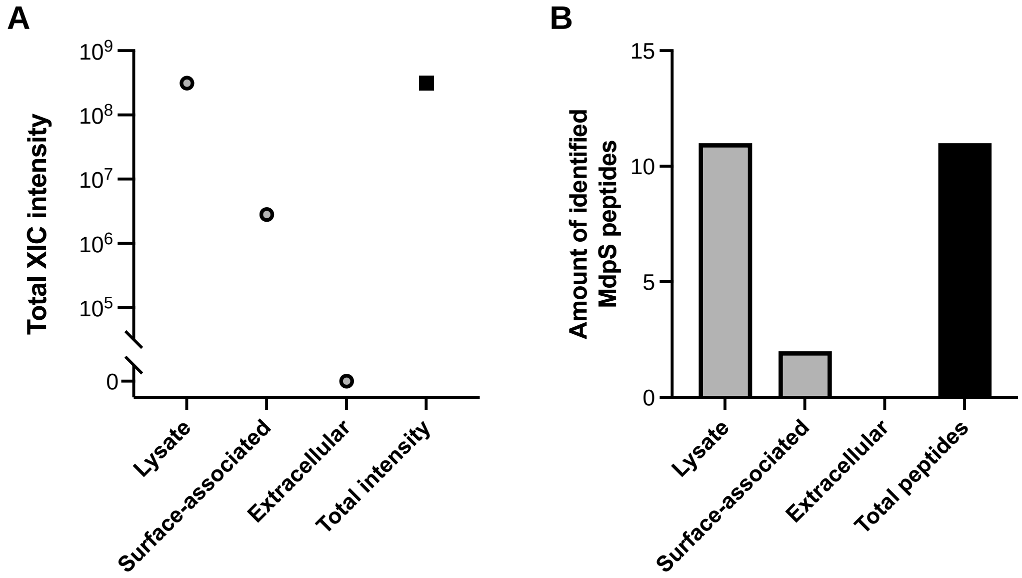

Supplementary figure 14: MdpS cellular localization in *Streptococcus oralis*. **(A)** The quantity in lysate, surface-associated or extracellular samples of *S. oralis* is based on the summed up eXtracted Ion Current (XIC) of all isotopic clusters associated with the identified MdpS amino acid sequence. **(B)** The total number of unique MdpS peptides are identified from the recorded tandem mass spectra.

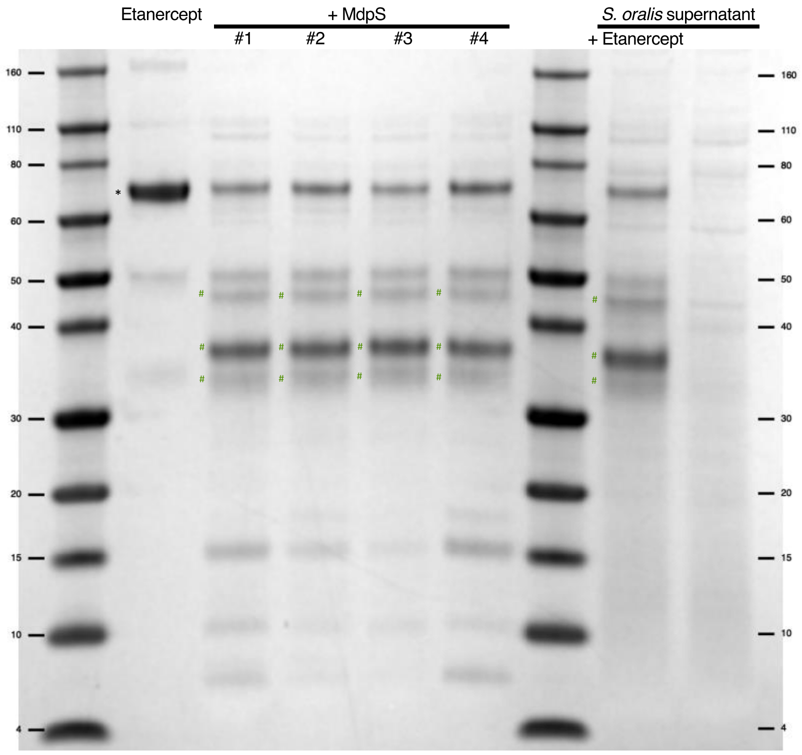

Supplementary figure 15: Activity of *S. oralis* supernatant towards Etanercept. The hydrolytic pattern of four MdpS replicates is compared with the *S. oralis* sample, analyzed with SDS-PAGE. Intact substrate (black asterisk: *), intact MdpS (green asterisk: *), and MdpS-generated fragments (green hashtag: #).
